# Comparative transcriptome analysis identified *ChlH* and *POLGAMMA2* in regulating yellow-leaf coloration in *Forsythia*

**DOI:** 10.3389/fpls.2022.1009575

**Published:** 2022-09-09

**Authors:** Man Zhang, Jianshuang Shen, Yutong Wu, Xiaolu Zhang, Zhengtian Zhao, Jia Wang, Tangren Cheng, Qixiang Zhang, Huitang Pan

**Affiliations:** ^1^Beijing Key Laboratory of Ornamental Plants Germplasm Innovation and Molecular Breeding, National Engineering Research Center for Floriculture, Beijing Laboratory of Urban and Rural Ecological Environment, College of Landscape Architecture, Beijing Forestry University, Beijing, China; ^2^Department of Landscape Architecture, Jiyang College, Zhejiang A&F University, Zhuji, China

**Keywords:** *Forsythia*, yellow-leaf, *ChlH*, *POLGAMMA2*, transcriptome sequencing, chlorophyll biosynthesis, chloroplast development

## Abstract

Leaf color is one of the most important features for plants used for landscape and ornamental purposes. However, the regulatory mechanism of yellow leaf coloration still remains elusive in many plant species. To understand the complex genetic mechanism of yellow-leaf *Forsythia*, we first compared the pigment content and leaf anatomical structure of yellow-leaf and green-leaf accessions derived from a hybrid population. The physiological and cytological analyses demonstrated that yellow-leaf progenies were chlorophyll deficient with defected chloroplast structure. With comparative transcriptome analysis, we identified a number of candidate genes differentially expressed between yellow-leaf and green-leaf *Forsythia* plants. Among these genes, we further screened out two candidates, *ChlH* (magnesium chelatase Subunit H) and *POLGAMMA2* (POLYMERASE GAMMA 2), with consistent relative-expression pattern between different colored plants. To verify the gene function, we performed virus-induced gene silencing assays and observed yellow-leaf phenotype with total chlorophyll content reduced by approximately 66 and 83% in *ChlH*-silenced and *POLGAMMA2-*silenced plants, respectively. We also observed defected chloroplast structure in both *ChlH*-silenced and *POLGAMMA2-*silenced *Forsythia*. Transient over-expression of *ChlH* and *POLGAMMA2* led to increased chlorophyll content and restored thylakoid architecture in yellow-leaf *Forsythia*. With transcriptome sequencing, we detected a number of genes related to chlorophyll biosynthesis and chloroplast development that were responsive to the silencing of *ChlH* and *POLGAMMA2*. To summarize, *ChlH* and *POLGAMMA2* are two key genes that possibly related to yellow-leaf coloration in *Forsythia* through modulating chlorophyll synthesis and chloroplast ultrastructure. Our study provided insights into the molecular aspects of yellow-leaf *Forsythia* and expanded the knowledge of foliage color regulation in woody ornamental plants.

## Introduction

The genus of *Forsythia* belongs to the family of *Oleaceae*, and is consisted of many deciduous species that are commonly used ornamental plants (Shen et al., 2019). *Forsythia* species are prized for their arching branches, golden blossoms, and early flowering features and are widely cultivated worldwide. The fruits of *Forsythia* were often used for medical treatment and herbal drug industries (Guo et al., 2007; Piao et al., 2008). During the cultivation history of *Forsythia*, many morphologically diverged cultivars were evolved and selected. *Forsythia koreana* “Suwon Gold” is a yellow-leaf cultivar, forming magnificent golden canopy throughout growing seasons (Wang et al., 2017). Comparing with green-leaf cultivars, *F. koreana* “Suwon Gold” is less vigorous and its leaves are more susceptible to sun burn during summer (Wang et al., 2017). Though great effort has been made to breed for colored leaf cultivars, the mechanism controlling yellow-leaf coloration in *Forsythia* remains unknown.

Leaf coloration is an important ornamental aspect for plants used in landscape applications. Various leaf color can enhance the ornamental effect of plant species and compensate for the monotonous color during growing period (Dong et al., 2020). Different leaf color often reflects the composition and content of major pigments, including chlorophyll, carotenoids, and flavonoids in the mesophyll cells (Sinkkonen et al., 2012; Yang et al., 2015). For example, the redness of red maple leaves is a result of low chlorophyll content and accumulated cyanidin (Chen et al., 2019). Chlorophyll and carotenoids are two major pigments that determine the leaf yellowing in plants (Luo et al., 2019; Mo-zhen et al., 2020). Previous studies have characterized more than twenty enzymatic steps required for chlorophyll biosynthesis (Sobotka, 2013). The first step is the insertion of Mg^2+^ into protoporphyrin IX (PP IX) to form Mg-PP IX, which is catalyzed by Mg-chelatase (Terry et al., 2002). Mg-chelatase is a protein complex that comprised of subunits encoded by *ChlI* (magnesium chelatase Subunit I), *ChlD* (magnesium chelatase Subunit D), and *ChlH* (magnesium chelatase Subunit H) (Wang and Grimm, 2015). Apart from catalyzing the chlorophyll branch, *ChlH* also participates in plastid-nuclear signaling pathway that regulate the expression of photosynthesis-related nuclear genes in *Arabidopsis* (Mochizuki et al., 2001). Mutations in *ChlH* gene led to underdeveloped thylakoid membrane and low chlorophyll content in rice (Jung et al., 2003). DVR encodes the 3,8-divinyl protochlorophyllide a 8-vinyl reductase that catalyzing another indispensable step for chlorophyll biosynthesis (Pogson et al., 2015). DVR mutants exhibited yellow-green phenotype with reduced chlorophyll content, arrested chloroplast development, and attenuated photosynthesis activity in rice (Rodríguez et al., 2016). Mutation in genes involved in chlorophyll degradation can also produce leaf color mutants (Zhao et al., 2020). For example, a yellow-leaf mutant of *Cymbidium sinense* is caused by the up-regulation of two enzymes, *CLH2* (CHLOROPHYLLASE 2) and *RCCR* (red chlorophyll catabolite reductase), which promote the breakdown of chlorophyll *a* (Amancio et al., 2015).

On the other hand, abnormal chloroplast development can also affect the chlorophyll accumulation and cause yellow leaf phenotype (Zhao et al., 2020). For example, the *Arabidopsis* “cue” mutants, exhibiting yellow-green and pale-green leaves, have delayed chloroplast differentiation and impaired chloroplast structure (Loìpez-Juez et al., 1998). Chloroplast development is regulated by the coordination of nuclear and chloroplast genes that mediate transcription and translation, thylakoid formation, pigment synthesis, plastid-nuclear signaling transduction, and chloroplast division (Pogson and Albrecht, 2011; Zhao et al., 2020). Any mutation in these genes may impair the chloroplast development and functioning (Kessler and Schnell, 2009). A genetic screen in yellow-leaf soybean detected a point mutation in *YL* (YELLOW LEAF), a chloroplast localized gene homologous to the *Arabidopsis ORRM1* (ORGANELLE RRM PROTEIN 1), which regulates chloroplast RNA-editing and photosynthesis (Zhu et al., 2020). In *Arabidopsis*, the mutation in *SCO1* (SNOWY COTYLEDON 1), which encodes the chloroplast elongation factor G, led to cotyledon albinism due to improperly developed chloroplasts (Ruppel and Hangarter, 2007). Despite the research advancements in leaf color regulation, the mechanism underlying leaf yellowing may differ across different plant species.

To understand the physiological basis of yellow leaf-color in *Forsythia*, we compared the pigment and anatomical characteristics of yellow-leaf and green-leaf accessions from a F_1_ hybrid population generated in a previous study (Wang et al., 2017). Subsequently, we performed comparative transcriptome analysis and obtained a number of differentially expressed genes that possibly related to the leaf color difference between yellow-leaf and green-leaf *Forsythia*. Among the candidate genes, we screened out two genes, *ChlH* and *POLGAMMA2*, with consistent expression patterns among different leaf-color progeny lines regardless of light-intensities. We further verified their functions with virus-induced gene silencing and transient over-expression technique in *Forsythia* and other model plant species. By analyzing the transcriptome change after gene silencing, we explored the possible genetic mechanism of *ChlH* and *POLGAMMA2* in regulating leaf-color in *Forsythia*. Our study revealed the physiological and transcriptional characteristics of yellow-leaf *Forsythia*, and provided valuable information for future breeding of colorful foliage plants.

## Materials and methods

### Plant materials

To reduce the genetical difference between two *Forsythia* species, we hybridized *F. koreana* “Suwon Gold” (♂) with *F*. “Courtaneur” (♀) and generated progenies with clear leaf-color variations (from green to yellow) in a previous study (Wang et al., 2017). All hybrids were planted in the nursery at National Engineering Research Center for Floriculture (40°02′ N, 115°50′ E) in Beijing, China. We randomly selected thirty progeny strains of yellow-leaf and green-leaf plants and propagated ten clones for each strain from stem cuttings. To study the effect of light intensity on leaf-coloration, we selected ten green-leaf (L1 group) and yellow-leaf (L2 group) lines with three biological replicates for each group, and cultivated them under the natural light conditions (daily light intensity range from 1,300 to 1,500 μmol⋅m^–2^⋅s^–1^). Similarly, we selected ten green-leaf (S1 group) and ten yellow-leaf accessions (S2 group) for shade treatment (daily light intensity 390–450 μmol⋅m^–2^⋅s^–1^). These two light-intensity treatments were performed from May 3, 2017 to June 3, 2017 with the same environmental temperatures. Mature leaves (the third and fourth leaves from the top) of new branches in each *Forsythia* plant were sampled for further analysis.

### Measurement of pigment content

To characterize the four treatment groups, we measured the color of mature leaves for plants in L1, L2, S1, and S2 group with the fifth edition Royal Horticultural Society Color Chart, respectively. Approximately 150 mg fresh leaves were collected for each sample and immersed into 20 ml of 80% acetone (v/v) at room temperature in the dark for 24 h. The absorbance of supernatant was measured for each group at 470 nm, 646, and 663 nm using BioMate 3S UV-visible spectrophotometer (Thermo Fisher Scientific, Shanghai, China) with three technical replicates. The chlorophyll (Chl *a*, Chl *b*, and total Chl) and carotenoid (Car) contents were determined according to Lichtenthaler (1987). The Chl/Car ratio was calculated as the total chlorophyll content to total carotenoids content.

### Leaf anatomy and chloroplast ultrastructure examination

Mature leaves were selected and fixed in FAA solution (acetic acid: 70% alcohol = 1:3). Cross sections (12 mm thick) were sliced using a microtome, stained with 50% water-soluble safranin and fast green, and mounted in gelatin-glycerine. For each leaf sample, five to ten sections were randomly selected for observation under microscope ZEISS Scope A1. The leaf thickness, thickness of upper epidermis, lower epidermis, palisade, and spongy parenchyma were measured using Image-Pro Plus 6.0. The CTR (cell tense ratio) and SR (spongy ratio) values were also calculated for three sections of three progeny lines randomly selected for each color group (He et al., 2017).

For transmission electron microscopy (TEM) analyses, mature leaves of each group were sampled and cut into 2 mm × 4 mm pieces fixed with 3% glutaraldehyde solution (glutaraldehyde solution is prepared with 0.1 mol/L phosphate buffer, pH 7.2) at 4°C for at least 2 hours. The samples were then rinsed with phosphate buffer for 3–5 times and fixed in 1% osmium tetroxide solution at 4°C overnight, followed by dehydration using gradient ethanol solutions (50, 70, 95, and 100% alcohol). Samples were embedded in epoxy resin and sliced with LEICA DC61 ultra-thin microtome (Leica Microsystems, Germany). The ultrathin sections were examined under JEM-1010 electron microscope (JEOL, Tokyo, Japan). For statistical analysis, we conducted at least three independent measurements for each parameter in ten mesophyll cells of three randomly selected leaves for each group. The leaf anatomical features and chloroplast structure were also examined and compared for the newly sprouted leaves of plants before and after transient silencing and over-expression of candidate genes.

### Transcriptome sequencing and candidate gene identification

Leaves were collected from ten randomly selected progeny lines of each color group (L1, S1, L2, S2) with three biological replicates. Total RNA was extracted and the RNA integrity was assessed using Agilent 2100 bioanalyzer. A total of twelve sequencing libraries were prepared and sequenced on Illumina Hiseq 2500 platform (Novogene Bioinformatics Technology Co., Ltd., Beijing, China). Raw reads were deposited in NCBI SRA archive as BioProject PRJNA437859 (SRA accession: SRS3039871-SRS3039882).

Raw reads were cleaned by filtering adaptor and low quality sequences with fastp v0.19.7 (Chen et al., 2018). We mapped all clean reads to the reference genome of *F. suspensa* (Li et al., 2020) using HISAT (Kim et al., 2015) and sorted the alignment with Samtools (Li et al., 2009). Transcript abundance level was estimated with Stringtie (Li and Dewey, 2011) and was normalized to FPKM value (Fragment Per kilo bases per Million reads). Differential expression analysis was performed on four groups using DEGseq R package (Wang et al., 2010). We defined genes with adjusted *p*-value ≤ 0.05 and fold change ≥2.0 as differentially expressed genes (DEGs). GO and KEGG enrichment analyses were performed on the DEGs using the “phyper” function in R. GO terms and KEGG pathways with FDR < 0.05 were considered as significantly enriched.

To determine the transcriptional profile established by the silencing of *FsChlH* and *FsPOLGAMMA2*, we performed RNA-seq on leaves of green-leaf *Forsythia* before and after *ChlH* and *POLGAMMA2* were transiently silenced, respectively. The procedure of sampling, RNA extraction, library construction, and sequencing were the same as described above. The raw sequence data generated correspondingly were deposited in NCBI SRA archive under BioProject PRJNA756703 (SRA accession: SRR15559813-SRR15559824). Differential expression analysis was conducted by comparing the *FsChlH*-silenced (CY) and *FsPOLGAMMA2*-silenced (PY) plants with their green-leaf vector controls (CG and PG). The DEGs were defined as genes with adjusted *p*-value ≤ 0.05 and fold change ≥2.0 in the differential expression analysis.

### Expression pattern analysis

To validate the results of RNA-seq and expression pattern of candidate genes, sample RNA was extracted with Plant RNA Kit (Omega Bio-Tek, United States) and was reverse transcribed using the Prime Script RT reagent Kit with gDNA Eraser (Takara Bio, Japan) following the manufacturer’s protocols. Quantitative real-time RT-PCR was performed using TB Green^®^ Premix Ex Taq II (Takara Bio, Japan) on CFX Connect Real-Time System (Bio-Rad Laboratories, Inc., United States) following the temperature setting: 30 s at 94°C, 40 cycles of 94°C for 5 s, 60°C for 30 s, and 72°C for 30 s. Relative gene expression was performed using the 2^–ΔΔCt^ method with *CYCLOPHILIN* gene used as reference gene (Shen et al., 2019). The primers of selected DEGs for qRT-PCR assays were designed with Primer Premier 5 software (Premier, United States) and were provided (Supplementary Table 1). Each qRT-PCR experiment was performed with three biological replicates and three technical replicates. qRT-PCR experiments were also performed to evaluate the candidate gene expression in plants with target genes transiently silenced or over-expressed. For model plant systems, *ACTIN* genes were used as internal reference for tomato (Chang et al., 2018) and tobacco (Aziz et al., 2019) in the qRT-PCR analysis.

### Plasmid construction

Full length sequence of *FsChlH* and *FsPOLGAMMA2* were cloned and were inserted into the pSuper1300 vector to generate the pSuper1300-*FsChlH* and pSuper1300*-FsPOLGAMMA2* for gene over-expression. Gene-specific fragments of *FsChlH* and *FsPOLGAMMA2* between 300 and 500 bp were cloned and transferred into the pTRV2 vector, which encodes the RNA2 genome of TRV (Tobacco Rattle Virus) (Fu et al., 2005). Primers used for preparing the expression vectors above were provided (Supplementary Table 2). All constructed vectors were sequencing confirmed by Sangon Biotech Inc. (Shanghai, China) and were transformed into the *Agrobacterium* GV3101 strain cells.

### Transient gene over-expression and silencing

For transient over-expression assay, the transformed agrobacteria with pSuper1300-*FsChlH* and pSuper1300*-FsPOLGAMMA2* were used to infect yellow-leaf plants following previously reported protocols (Yamamoto et al., 2018). With leaf veins as the boundary, we infected only one side of the leaf, leaving the other side as the control. For VIGS assay, the green-leaf *Forsythia* plants were infiltrated with *Agrobacterium* strains containing the mixture of pTRV2 and pTRV2-*FsChlH*, pTRV2, and pTRV2-*FsPOLGAMMA2*, with empty-loaded pTRV2 as the mock based on previously established protocol (Shen et al., 2021). For each treatment, six individual plants were infected, kept in dark for 24 h, and were maintained in climatic chamber [light intensity 300 μmol⋅m^–2^⋅s^–1^; day length: 16 h (22°C)/8 h (18°C); relative humidity 60%]. Similarly, VIGS studies were performed in tomato (*Solanum lycopersicum*) and tobacco (*Nicotiana benthamiana*) following the standard protocols (Senthil-Kumar and Mysore, 2011). The plants with transient silencing or over-expression of target genes were examined for leaf pigment content and chloroplast structure following same protocols described above.

### Statistical analysis

All data were analyzed using SPSS software v.17.0 and the statistical significances of leaf pigment contents, anatomical features, and gene expression levels were assessed using one-way ANOVA followed by multiple comparisons using Duncan’s Multiple Range Test (DMRT).

## Results

### Phenotypic analysis of yellow-leaf *Forsythia*

**FIGURE 1:**
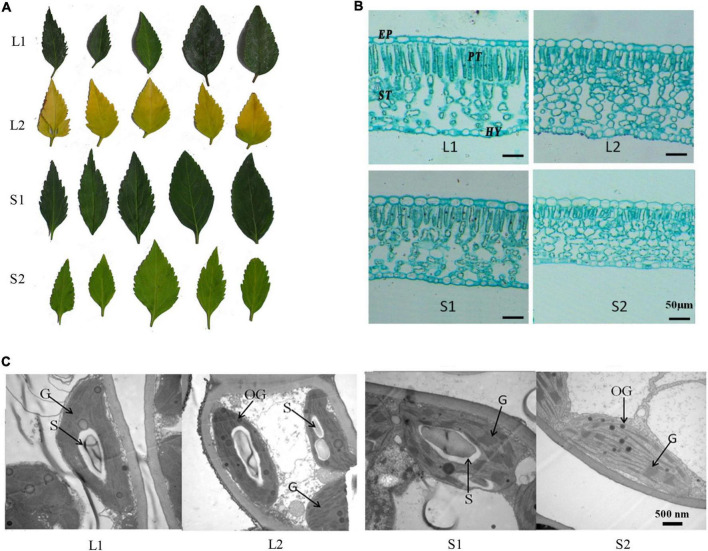
Phenotypic analysis of yellow-leaf and green-leaf *Forsythia* plants under different light-intensity treatments. **(A)** Leaf color of four groups (L1: green-leaf *Forsythia* under natural light condition; L2: yellow-leaf *Forsythia* under natural light condition; S1: green-leaf *Forsythia* under shade condition; S2: yellow-leaf *Forsythia* under shade condition). **(B)** Leaf anatomy of plants in four groups. EP refers to epidermis; PT refers to palisade tissue; ST refers to spongy tissue; HY refers to Hypodermis. **(C)** Chloroplast ultrastructure of plants in four groups. G refers to granum; S refers to starch grain; OG refers to osmiophilic globule.

The yellow-leaf progenies with yellowish leaf (YG11A-YGGN144A) under natural light conditions convert to yellow-green (YGG144A-C) when transferred to low light-intensity condition (Figure 1A). While the virescent leaves of green-leaf lines (YGG146A-B) converted to green (GG137A-GGN137C) after shade treatment (Figure 1A). The pigment content analysis revealed that Chl *a*, Chl *b*, total Chl content, and carotenoid of yellow-leaf lines under natural light conditions (L2) were 0.12, 0.26, 0.38, and 0.29 mg/g, respectively (Table 1). In contrast, the Chl *a*, *b*, and carotenoid content in green-leaf lines (L1) were 0.77, 0.29, and 0.61 mg/g, respectively (Table 1). After the shading treatment, chlorophyll and carotenoid contents were both significantly increased in green-leaf and yellow-leaf plants (Table 1). The Chl/Car ratio in yellow-leaf plants after shading treatment is significantly higher than that of the yellow-leaf plants under normal daylight condition (Table 1). However, there is no significant differences in Chl/Car ratio between yellow-leaf and green-leaf plants under natural light conditions (Table 1). We observed significantly higher level of Chl *a* in green-leaf lines than that of yellow-leaf lines in both natural light and shade conditions. Moreover, no significant difference was observed in Chl *b* content among the four treatment groups (Table 1). Therefore, the accumulation of Chl *a* content is likely one major cause for the leaf-color difference between green-leaf and yellow-leaf *Forsythia* lines.

**TABLE 1:** The photosynthetic pigment content in green-leaf and yellow-leaf plants under different light intensities.

Group	Chl *a* (mg/g)	Chl *b* (mg/g)	Total chlorophyll (mg/g)	Carotenoid (mg/g)	Ratio of Chl/Car
L1	0.77 ± 0.05^c^	0.29 ± 0.11^a^	0.93 ± 0.16^b^	0.61 ± 0.07^c^	1.73 ± 0.05^ab^
L2	0.12 ± 0.05^a^	0.26 ± 0.17^a^	0.38 ± 0.12^a^	0.29 ± 0.04^a^	1.26 ± 0.48^a^
S1	1.22 ± 0.11^d^	0.48 ± 0.11^a^	1.69 ± 0.19^c^	0.88 ± 0.08^d^	1.93 ± 0.06^ab^
S2	0.45 ± 0.11^b^	0.44 ± 0.10^a^	0.89 ± 0.20^b^	0.43 ± 0.03^b^	2.1 ± 0.28^b^

Total chlorophyll content = Chl a + Chl b; Ratio of Chl/Car = total chlorophyll content/total carotenoids content; For different pigment contents within each column, mean ± se followed by the same alphabetic letters are not significantly different in Duncan’s Multiple Range test under significance level 0.05.

To compare the leaf anatomical features in four treatment groups, we measured the thickness of four tissue types: epidermis (EP), palisade tissue (PT), sponge tissue (ST), hypoderm (HY). The thickness of EP, PT, and CTR (Palisade thickness/Leaf thickness) of yellow-leaf *Forsythia* plants (L2 and S2) were significantly lower than those of green-leaf *Forsythia* plants (L1 and S1) (Supplementary Table 3 and Figure 1B). After shade treatment, the sponge tissue thickness in both yellow-leaf (L2) and green-leaf *Forsythia* plants (L1) decreased dramatically (Supplementary Table 3 and Figure 1B). By examining the chloroplast structure with TEM, we observed abnormal chloroplast structure in yellow-leaf plants (Figure 1C). Under natural light conditions, the chloroplasts of the green-leaf *Forsythia* plants (L1) were well-developed with normally stacked grana (Figure 1C). Each chloroplast contains 1–2 huge starch grains and a few plastoglobules. However, yellow-leaf *Forsythia* plants (L2) contained chloroplasts lacking grana with only abnormally arranged lamellar structures (Figure 1C and Supplementary Table 4). After shading treatment, the chloroplasts of green-leaf plants (S1) remain normal, while a few loosely packed thylakoids with few layers were visible in the chloroplasts of yellow-leaf (S2) *Forsythia* plants (Figure 1C and Supplementary Table 4). Moreover, the number of osmiophilic globules was significantly increased in yellow-leaf *Forsythia* plants than that of green-leaf lines in shade condition (Figure 1C and Supplementary Table 4). These results suggested that the chloroplast development and chlorophyll biosynthesis were disrupted in the yellow-leaf *Forsythia* plants and these defects cannot be fully recovered in low irradiance condition.

### Transcriptome sequencing and differentially expressed gene analysis

To understand the molecular mechanism of yellow leaf coloration in *Forsythia*, we performed RNA-seq on leaves of yellow-leaf and green-leaf plants. After trimming adapters and filtering out low-quality reads, we obtained a total of 120.22 Gb clean reads generated from twelve sequencing libraries (Supplementary Table 5). With differential expression analysis, we identified a total of 1820 DEGs between yellow-leaf and green-leaf *Forsythia* lines under natural light conditions and 346 DEGs after shade treatment (Supplementary Figure 1). By comparing the DEG sets from high and low light-intensity treatments, we obtained 157 common DEGs. To validate the accuracy of RNA-seq results, we performed qRT-PCR analysis and observed that the relative gene expression pattern of the selected DEGs is in agreement with that in transcriptome data (Supplementary Figure 2). Among the DEGs identified in normal light condition, 1011 DEGs were up-regulated and 809 DEGs were down-regulated. Functional enrichment analysis revealed that these DEGs were significantly enriched in oxidation-reduction process (GO:0055114), carbohydrate metabolic process (GO:0005975), and DNA repair (GO:0006281) (Supplementary Table 6). Among the DEGs identified between yellow-leaf and green-leaf lines after shade treatment, we identified 127 up-regulated DEGs and 219 down-regulated DEGs. These DEGs were found mainly involved in biological processes, such as nucleic acid-templated transcription (GO:0097659), galactose metabolic process (GO:0006012), and oxidation-reduction process (GO:0055114) (Supplementary Figure 1 and Supplementary Table 6).

### Identification of candidate genes related to yellow-leaf coloration in *Forsythia*

**FIGURE 2:**
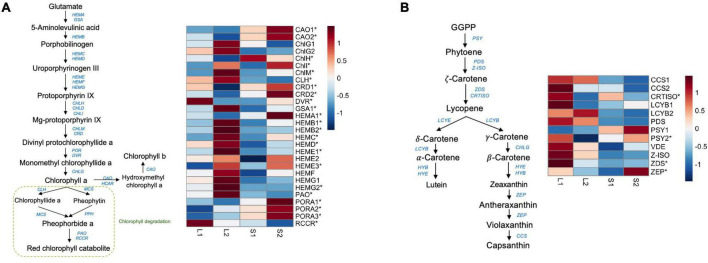
The expression profile of genes involved in chlorophyll and carotenoid biosynthesis. **(A)** The candidate genes in the chlorophyll biosynthetic pathway and their expression profiles across four treatment groups; **(B)** the candidate genes in the carotenoid biosynthetic pathway and their expression profiles across four treatment groups. The star symbols highlight the DEGs in either group comparisons.

To screen for candidate genes associated with yellow-leaf phenotype, we mainly focused on analyzing the expression profiles of DEGs involved in pigment biosynthesis and chloroplast development and only considered those with consistent relative expression pattern among different leaf-color plants for downstream functional verification. In total, we identified 19 DEGs related to chlorophyll biosynthesis and three DEGs related to carotenoid biosynthesis (Supplementary Table 7). We observed the down-regulation of *ChlH*, *CRDs* (COPPER RESPONSE DEFECT 1), *DVR*, and *HEMA1* (glutamyl-tRNA reductase) in the yellow-leaf plants comparing to green-leaf lines, which is consistent with the low chlorophyll content in yellow-leaf plants under natural light conditions (Figure 2A). In contrast, a few genes promoting the biosynthesis of Chl *a*, such as *ChlM* (magnesium protoporphyrin IX methyltransferase), *CHlI* (magnesium chelatase subunit I), *GSA1* (GLUTAMATE-1-SEMIALDEHYDE-2,1-AMINOMUTASE), and *HEMBs-HEMGs* displayed higher expression level in yellow-leaf plants (Figure 2). *CAO2* (CHLOROPHYLL A OXYGENASE), the key enzyme involved in Chl *b* biosynthesis, also showed lower expression level in yellow-leaf plants (Figure 2). We also observed a number of DEGs displaying contrasting expression patterns between yellow-leaf and green-leaf plants after shade treatment (Figure 2A). For example, the transcription levels of *CAO1*-*2*, *CRD1-2*, *DVR*, *HEMA1*, and *PORA1*-3 (PROTOCHLOROPHYLLIDE OXIDOREDUCTASE A) were low in yellow-leaf plants, but were strongly up-regulated under low light-intensity condition (Figure 2A). Among all the DEGs related chlorophyll biosynthesis, *ChlH* were consistently down-regulated in yellow-leaf lines regardless of the light-intensity condition (Figure 2A). Thus, we considered *ChlH* as one of the key candidate genes for the following functional analysis.

We also detected four DEGs that were involved in carotenoid synthesis (Supplementary Table 7). These include *PSY2* (PHYTOENE SYNTHASE), *CRTISO* (CAROTENOID ISOMERASE), *ZEP* (ZEAXANTHIN EPOXIDASE), and *ZDS* (ZETA-CAROTENE DESATURASE). All four genes were relatively down-regulated in yellow-leaf lines under natural light conditions (Figure 2B). However, the expression of *PSY2* and *ZEP* was significantly induced in shade condition (Figure 2B). *ZDS*, on the other hand, remained lowly expressed in both green-leaf and yellow-leaf *Forsythia* plants after shade treatment (Figure 2B). *CRTISO* was consistently down-regulated in yellow-leaf plants under both natural-light and shade conditions (Figure 2B). Since the content level of carotenoids is not the major reason for yellow leaf-color, we excluded the candidate genes related to carotenoid biosynthesis from further functional investigations.

Furthermore, we examined the expression pattern of DEGs that are related to photosynthesis, chlorophyll binding and chloroplast development. Among them, three genes were annotated to lipid metabolism, including *GLTP3* (GLYCOLIPID TRANSFER PROTEIN 3), *LTPL101* (lipid-transfer family protein), and *GDPD3* (GLYCEROPHOSPHODIESTER PHOSPHODIESTERASE 3). The expression levels of *GLTP3* and *GDPD3* were significantly higher in yellow-leaf plants, but were then dropped in shading condition (Supplementary Figure 3). The expression of *LTPL101* was low in natural light conditions, and were induced in both green and yellow plants after shading treatment (Supplementary Figure 3). We observed the transcriptional pattern change of photosynthesis-related DEGs as the irradiance change. For example, *PsaAs* (PHOTOSYSTEM I SUBUNIT A), *PsbAs* (PHOTOSYSTEM II REACTION CENTER PROTEIN A), and *PsbBs* (PHOTOSYSTEM II REACTION CENTER PROTEIN B) were up-regulated in yellow-leaf plants (Supplementary Figure 3). *PsaHs* (PHOTOSYSTEM I SUBUNIT H2), *PsbYs* (PHOTOSYSTEM II BY), and *PsbP2* (PHOTOSYSTEM II SUBUNIT P) were relatively up-regulated in green-leaf plants, but their expression levels were higher in yellow-leaf plants after transferred to shade condition (Supplementary Figure 3). Additionally, we characterized a few candidate genes, such as *PTAC3* (PLASTID TRANSCRIPTIONALLY ACTIVE 3) and *POLGAMMA2*, that putatively regulate chloroplast development. PTAC3 is an essential component of PEP complex (plastid encoded RNA polymerase) that regulate the photosynthetic gene expression, chloroplast development and functioning (Kindgren and Strand, 2015). The expression of *PTAC3* was high in yellow-leaf plants under natural light, but it significantly decreased in shading condition (Supplementary Figure 3). *POLGAMMA2* encodes a nuclear-encoded organelle DNA polymerase targeting to mitochondria and plastids (Baruch-Torres and Brieba, 2017). The expression of *POLGAMMA2* was relatively low in yellow-leaf *Forsythia* lines under both natural light and shading conditions, showing relative expression pattern consistent with leaf color change among samples. Therefore, we also tested the function of *POLGAMMA2* with the following functional assays.

### Virus-induced silencing of *ChlH* and *POLGAMMA2* in *Forsythia*

**FIGURE 3:**
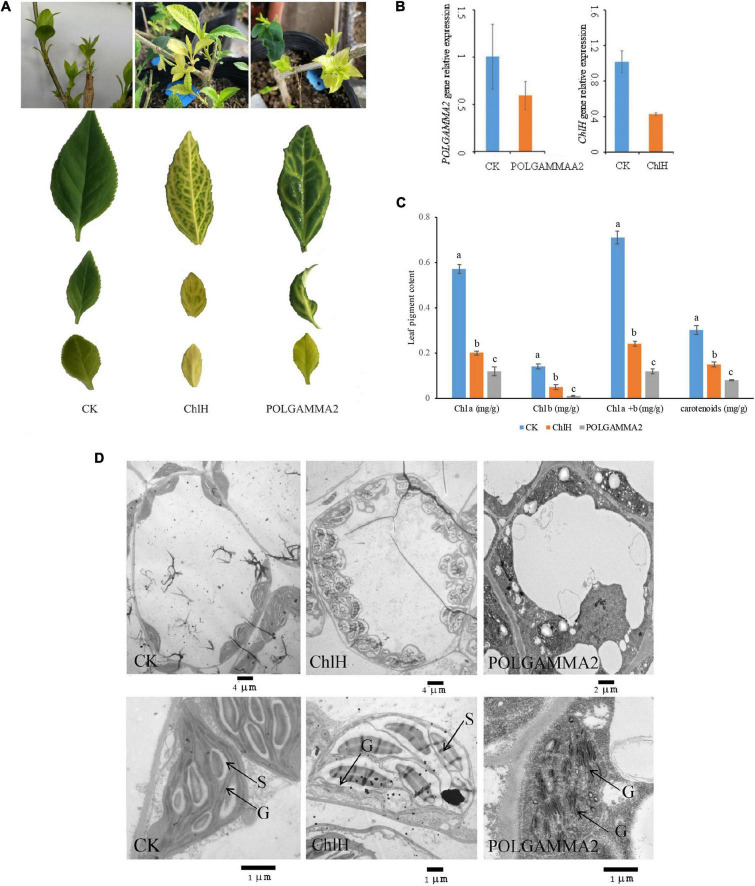
The leaf color and anatomy change in green-leaf *Forsythia* plants with candidate genes transiently silenced. **(A)** The leaf color of CK (vector control), *FsChlH*-silenced, and *FsPOLGAMMA2*-silenced plants; **(B)** the relative gene expression of *POLGAMMA2* and *ChlH* in *FsPOLGAMMA2*-silenced and *FsChlH*-silenced plants, respectively; **(C)** the content of Chl *a*, Chl *b*, total Chl, and carotenoid in CK, *FsChlH*-silenced, and *FsPOLGAMMA2*-silenced *Forsythia* plants; **(D)** the chloroplast ultrastructure of CK, *FsChlH*-silenced, and *FsPOLGAMMA2*-silenced plants.

To further elucidate the function of *ChlH* and *POLGAMMA2*, we performed VIGS assays in green-leaf *Forsythia* plants (Figure 3). We observed normal green colored leaves and shoots regenerated from the *Forsythia* injected with empty-loaded TRV2 (vector control). However, leaves of plants expressing *TRV*:*FsChlH* and *TRV:FsPOLGAMMA2* showed bright yellow-leaf phenotype (Figure 3A). The younger leaves displayed bright yellow-color in the silenced plants, while the mature leaves showed variegated leaves (Figure 3A). We measured the relative expression of *ChlH* and *POLGAMMA2* in the newly generated shoots from *FsChlH*-silenced and *FsPOLGAMMA2*-silenced plants at 15 days post the inoculation. qRT-PCR analysis showed that *ChlH* and *POLGAMMA2* were significantly down-regulated in the *FsChlH*- and *FsPOLGAMMA2*-silenced plants, respectively (Figure 3B). We also observed significantly reduced chlorophyll and carotenoid content in the silenced plants (Figure 3C and Supplementary Table 8). The total chlorophyll decreased about 66.2 and 83.1% in *FsChlH*-silenced and *FsPOLGAMMA2*-silenced *Forsythia*s, respectively (Supplementary Table 8). Moreover, we observed slightly increased number of chloroplasts with fewer grana stacks in *FsChlH*-silenced *Forsythia* plants (Figure 3D and Supplementary Table 9). In *FsPOLGAMMA2*-silenced plants, the thylakoid membrane system was defective with improperly organized granum structures (Figure 3D and Supplementary Table 9). The number of starch grains showed no difference between *FsChlH*-silenced and the control, but was significantly dropped in *FsPOLGAMMA2*-silenced plants (Supplementary Table 9).

### Virus-induced silencing of *ChlH* and *POLGAMMA2* in tobacco and tomato

**FIGURE 4:**
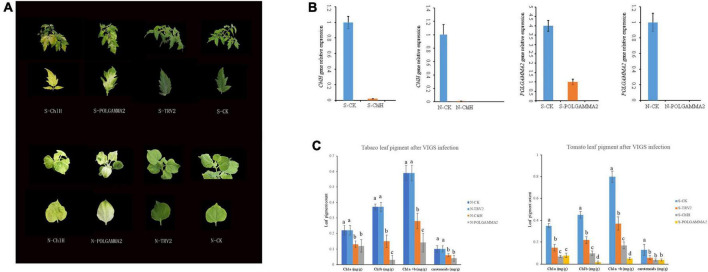
The leaf color change in tobacco and tomato plants after transiently silencing candidate genes. **(A)** The leaf color of *ChlH*-silenced (S/N-ChlH), *POLGAMMA2*-silenced (S/N-POLGAMMA2), TRV2 (vector control; S/N-TRV2), and non-infected (S/N-CK) tomato (S-) and tobacco (N-) plants. **(B)** The relative gene expression of *ChlH* and *POLGAMMA2* in *ChlH*-silenced and *POLGAMMA2*-silenced tomato (S-) and tobacco (N-) plants, respectively. **(C)** The content of Chl *a*, Chl *b*, total Chl, and carotenoid in CK, TRV2, *ChlH*-silenced, and *POLGAMMA2*-silenced tomato (S-) and tobacco (N-) plants.

To test if the functional role of *ChlH* and *POLGAMMA2* are conserved in other plant species, we used VIGS technique to reduce the transcription level of *ChlH* and *POLGAMMA2* gene in tobacco (*Nicotiana benthamiana*) and tomato (*Solanum lycopersicum*). We observed similar leaf color change in *ChlH*- and *POLGAMMA2-*silenced plants (Figure 4A). The newly sprouted leaves from *ChlH*-silenced tobacco (N-*ChlH*) and tomato (S-*ChlH*) showed yellow-color at 20 days after inoculation. The leaf color of *POLGAMMA2-*silenced tobacco (N-*POLGAMMA2*) and tomato (S-*POLGAMMA2*) were faded into white color (Figure 4A). The expression of *ChlH* and *POLGAMMA2* was barely detected in tomato and tobacco leaves expressing *TRV:ChlH* and *TRV:POLGAMMA2*, respectively (Figure 4B). Additionally, both chlorophyll and carotenoid content were significantly reduced with the down-regulation of *ChlH* and *POLGAMMA2* in tomato and tobacco leaves (Figure 4C and Supplementary Tables 10, 11). The total chlorophyll decreased by 53 and 54% in *ChlH*-silenced tobacco and tomato, and by 76 and 86% in *POLGAMMA2-*silenced tobacco and tomato, respectively (Supplementary Tables 10, 11). In *ChlH*-silenced tomato and tobacco plants, the width of chloroplasts was reduced by approximately 30% and the number of chloroplasts was reduced by 20% (Supplementary Tables 12, 13). We observed loosely packed grana with fewer layers of thylakoids in *ChlH*-silenced tomato and tobacco plants (Supplementary Figure 4). In *FsPOLGAMMA2*-silenced plants, the thylakoid membrane system was severely disrupted with no obvious granum structures observed (Supplementary Figure 4 and Supplementary Tables 12, 13).

### Transient expression of *ChlH* and *POLGAMMA2* genes in *Forsythia*

**FIGURE 5:**
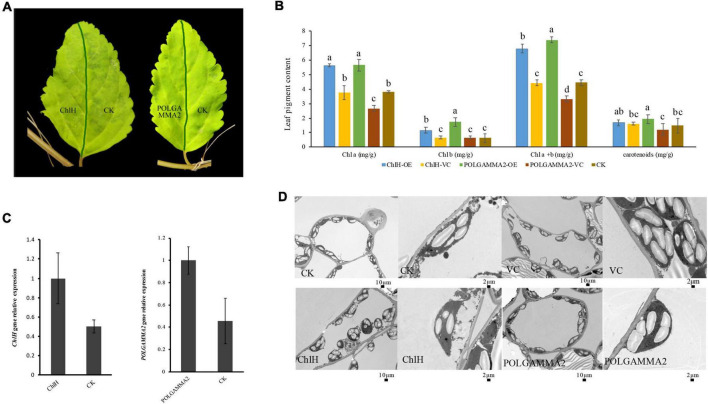
The leaf color and anatomy change in yellow-leaf *Forsythia* plants with candidate genes transiently over-expressed. **(A)** The leaf color of *ChlH*-OE, *POLGAMMA2*-OE, and the non-infected (CK) leaves of *Forsythia*. **(B)** The content of Chl *a*, Chl *b*, total Chl, and carotenoid in *ChlH*-OE, *POLGAMMA2*-OE, vector control, and the non-infected plants. **(C)** The relative gene expression of *ChlH* and *POLGAMMA2* in *ChlH*-OE and *POLGAMMA2*-OE plants, respectively. **(D)** The chloroplast structure of CK, VC (vector control), *ChlH*-OE, and *POLGAMMA2*-OE plants.

To further examine if the expression of *ChlH* and *POLGAMMA2* can rescue the leaf-color, we performed transient over-expression of these two genes in yellow-leaf *Forsythia* plants. With qRT-PCR assays, we found that the expression of *ChlH* and *POLGAMMA2* in the over-expression plants were twice as high as that of the control (Figure 5C). However, no obvious leaf color change was observed after the transient over-expression of *FsChlH* and *FsPOLGAMMA2* at 20 days post inoculation (Figure 5A). We also detected significantly increased content of Chl *a* and Chl *b* in plants with *ChlH* and *POLGAMMA2* transiently over-expressed (Figure 5B and Supplementary Table 14). The total chlorophyll content of yellow-leaf plants increased about two-fold after transient over-expressing *ChlH* and *POLGAMMA2*, while carotenoids remained unchanged in *ChlH* and *POLGAMMA2* over-expressed leaves (Figure 5B and Supplementary Table 14). Though the grana structures remained defected in plants over-expressing *ChlH* or *POLGAMMA2*, we observed some lamellae structures of thylakoids in plants over-expressing *POLGAMMA2* (Figure 5D and Supplementary Table 15).

### Identification of genes responsive to the silencing of *ChlH* and *POLGAMMA2*

**FIGURE 6:**
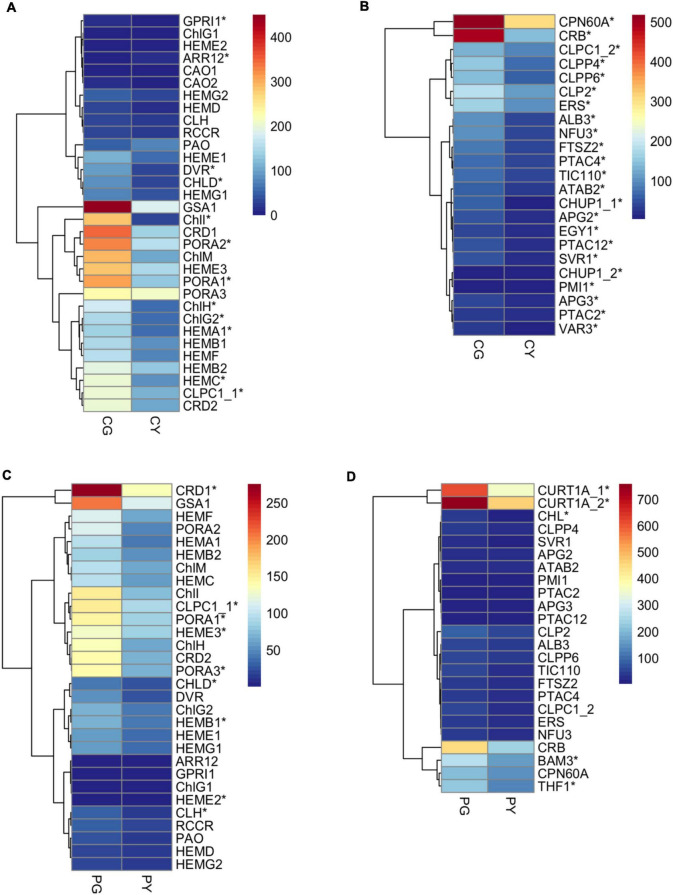
The expression pattern of key genes related to chlorophyll biosynthesis and chloroplast development in the transiently silenced *Forsythia* plants. **(A)** DEGs related to chlorophyll biosynthesis in the comparison of CG vs. CY; **(B)** DEGs related to chloroplast development in the comparison of CG vs. CY; **(C)** DEGs related to chlorophyll biosynthesis in the comparison of PG vs. PY; **(D)** DEGs related to chloroplast development in the comparison of PG vs. PY.

To understand the mechanism of yellow-leaf phenotype in *FsChlH*- and *FsPOLGAMMA2*-silenced plants, we performed transcriptome sequencing on leaves collected from the agroinfiltrated plants with *TRV:ChlH* (CY), *TRV:POLGAMMA2* (PY), and the vector control plants (CG and PG). In total, we identified 2040 DEGs between CY and CG, and 77 DEGs between PY and PG. The DEGs that are responsive to the decreasing level of *ChlH* are enriched in biological processes including photosynthesis (GO:0015979), chloroplast organization (GO: 0009658), response to light stimulus (GO:0009416), chlorophyll biosynthetic process (GO:0015995), and carotenoid biosynthetic process (GO:0016117) (Supplementary Table 16). In the *ChlH*-silenced plants, we observed decreased level of *ChlI* and *ChlD*, which encode two subunits of magnesium chelatase that catalyzing the insertion of Mg^2+^ into protoporphyrin IX in the first step of chlorophyll biosynthesis (Figure 6A). The transcription level of other chlorophyll biosynthesis genes (*HEMC*, *HEMAs*, *DVR*, and *PORAs*) were also reduced significantly with the decreasing level of *ChlH* (Figure 6A). Additionally, a few chloroplast development related genes were found significantly repressed in *ChlH*-silenced plants, including *PTACs* (plastid encoded RNA polymerases), *CPN60A* (CHAPERONIN-60ALPHA) that involved in Rubisco folding, *CRB* (CHLOROPLAST RNA BINDING), *CLPP4* (CLP PROTEASE P4), and *CLPP6* (CLP PROTEASE P6) that involved in chloroplast organization, *TIC110* (TRANSLOCON AT THE INNER ENVELOPE MEMBRANE OF CHLOROPLASTS 110) that regulate chloroplast biogenesis (Chen and Li, 2017), and *ALB3* (ALBINO 3) that regulate thylakoid membrane organization (Figure 6B). Moreover, we observed reduced transcription of carotenoid biosynthesis genes, such as *PSY2*, *LYC* (LYCOPENE CYCLASE), and *ZDS*, and a few carotenoid degradation genes including *CYP97A3* (CYTOCHROME P450-TYPE MONOOXYGENASE 97A3) and *BETA-OHASE 1* (BETA-HYDROXYLASE 1) in the *ChlH*-silenced plants (Supplementary Figure 5A). The expression of genes that involved in the photosynthesis were also found decreased, including the PHOTOSYSTEM I (*PsaAs*, *PsaBs*, *PsaG*, *PsaL*, *PsaO*) and PHOTOSYSTEM II subunits (*PsbB2*, *PsbP2*, *PsbE*, *PsbCs*, *PsbRs*), *LHCA1s* (PHOTOSYSTEM I LIGHT HARVESTING COMPLEX GENE 1), and *LHB1B1s* (LIGHT-HARVESTING CHLOROPHYLL-PROTEIN COMPLEX II SUBUNIT B1) (Supplementary Figure 5B).

Genes with drastic expression change after silencing of *FsPOLGAMMA2* are mostly involved in biological processes including phenylpropanoid metabolic process (GO:0009698), response to oxidative stress (GO: 0006979), and cellular lipid metabolic process (GO: 0044255) (Supplementary Table 17). The silencing of *POLGAMMA2* led to reduced transcript levels of chlorophyll synthetic genes, such as *ChlI*, *ChlH*, *PORA1*, *PORA3*, *HEME3*, *CRD1*, and carotenoid biosynthetic gene such as *PSY2*, *CCS2* (plastid encoded RNA polymerase), and *ZEP* (Figure 6C and Supplementary Figure 5C). Additionally, the genes related to chlorophyll binding and photosynthesis, including *PsaG*, *PsaH*, *PsaL*, *PsbO2*, *PsbP2*, *PsbR1*, *PsbYs*, *LHB1B1*, and *LHCAs*, were also significantly reduced (Supplementary Figure 5D). Moreover, we observed strong suppression of thylakoid development genes, including the *CHL* (CHLOROPLASTIC LIPOCALIN) that protects thylakoidal membrane lipids (Levesque-Tremblay et al., 2009), *THF1* (THYLAKOID FORMATION1) that involved in the vesicle-mediated formation of thylakoid membrane (Ma et al., 2015), and *CURT1A* (CURVATURE THYLAKOID 1A) that is essential for thylakoid granum assembly (Armbruster et al., 2013; Figure 6D). The reduced transcription of genes related to pigment biosynthesis and chloroplast development may lead to the yellow-leaf phenotype in plants with *FsChlH* and *FsPOLGAMMA2* gene silenced.

## Discussion

### Physiological and cytological basis of yellow-leaf coloration in *Forsythia*

Leaf coloration is an important attribute for ornamental plants. The diversity of leaf color can enhance the ornamental effect of landscape and extend the duration (Dong et al., 2020). Chlorophyll, carotenoid, and flavanols are three major types of pigments that determines the foliage color in plants (Zhao et al., 2020). So far, leaf color mutants have been characterized in many plant species, such as *Arabidopsis* (Jarvis et al., 1998), rice (Khan et al., 2021), wheat (Zhang et al., 2020), poplar (Ramamoorthy et al., 2019), and tomato (Mo-zhen et al., 2020). Yellow-leaf phenotype is often the direct consequence of changed contents in chlorophyll and carotenoids (Dong et al., 2020). For example, previous study reported reduced chlorophyll and increased lutein in golden-leaf gingko mutant (Li et al., 2018). Another study in *Populus deltoids* Marsh revealed that the disruption in chlorophyll synthesis or catabolism lead to lower level of chlorophyll and carotenoids in the yellow-leaf mutants (Ramamoorthy et al., 2019). In addition to pigment abundance, defective chloroplast development and ultrastructure can also impact leaf color (Dong et al., 2020). A spontaneous yellow-green leaf color mutant in *Triticum aestivum* L. exhibited reduced chlorophyll content and abnormal chloroplast development (Wu et al., 2018). In the leaf-color mutants of *Anthurium andraeanum* “Sonate,” the number of chloroplasts was significant reduced with ruptured chloroplasts in leaf mesophyll cells (Yang et al., 2015).

In our study, we have examined the physiological and anatomic characteristics of yellow-leaf *Forsythia* lines. We found that the yellow-leaf is determined by the combined effects of chlorophyll deficiency and disrupted chloroplast structure. Previous studies have demonstrated that different light intensities can affect the synthesis/degradation of photosynthetic pigments, the chloroplast development, and organelle structure (Kong et al., 2016). Upon light exposure, chlorophylls are synthesized, photo-oxidized and degraded rapidly (Zhu et al., 2017). Excessive light usually leads to plants with decreased chlorophyll content due to inhibited chloroplast formation and reduced thylakoid membrane appression (Fu et al., 2012). On the contrary, plants grown in shady environment acclimate and develop larger, thinner foliage with increased chlorophyll production and more appressed thylakoids to optimize the energy usage and conservation (Middleton, 2001; Anderson et al., 2008). Long-term deficient light can lead to degradation of chlorophyll, resulting in yellow leaves (Lee et al., 2014). Thus, we also evaluated the leaf anatomy and chloroplast ultrastructure of progeny lines grown in low light-intensity conditions. Both yellow-leaf and green-leaf lines displayed thinner leaves and palisade tissues after shade treatment, a phenomenon commonly observed in other plant species (Kong et al., 2016). Despite severely distorted grana, a few thylakoid structures were observed in the yellow-leaf plants grown under shady environment with chlorophyll content significantly increased, suggesting the thylakoid membrane appression and chlorophyll synthesis may be slightly enhanced when exposed to low irradiance condition. However, the low light intensity is not sufficient to restore the defected thylakoid membrane system and recover leaf-greening in the yellow-leaf *Forsythia* plants. Additionally, the photosynthetic capacity of the hybrid population was assessed previously by Wang et al. (2017). The photosynthetic performance of yellow-leafed plants is generally weaker than that of green-leafed plants. The reduced photochemical efficiency may be due to incomplete photosynthetic system on the underdeveloped chloroplast thylakoid development in yellow-leaf *Forsythia* plants (Wang et al., 2017). These results suggested that the deficiency in chlorophyll content and distorted chloroplast structure are likely two major causes of yellow-leaf coloration in *Forsythia* independent of light intensity.

### Molecular mechanism underlying yellow-leaf coloration in *Forsythia*

The formation of yellow-leaf is controlled by complex genetic network and environmental factors. Any mutations in genes related to pigment synthesis and metabolism, chloroplast transcription, and plastid-nuclear signal transduction can directly or indirectly disrupt pigment biosynthesis and stability, resulting in leaf discoloration (Zhao et al., 2020). In previous studies, RNA-seq approach has been widely applied to investigate candidate genes related to yellow-leaf coloration in plants (Zhao et al., 2020). For instance, the transcriptome study on golden leaf ginkgo mutants revealed that the down-regulated chlorophyll biosynthetic gene as well as up-regulated genes related to chlorophyll degradation and carotenoid biosynthesis likely caused lower chlorophyll and higher carotenoid detected in yellow-leaf mutants (Li et al., 2018). Similar findings were reported in yellow-leaf Shumard oak (Dong et al., 2020). A comprehensive study of yellow-leaf mutant of *Lagerstroemia indica* identified eleven candidates involved in chlorophyll metabolism, photosynthesis, and chloroplast development that may be responsible for altered chlorophyll content level and hindered chloroplast development in the mutants (Li et al., 2015).

In order to understand the molecular basis of yellow-leaf phenotype, we performed transcriptome sequencing on leaves of yellow-leaf and green-leaf *Forsythia* hybrids grown under different light-intensity conditions. Based on the comparative transcriptome analysis, we identified a large number of DEGs that were annotated to different biological processes including carbohydrate metabolic process, oxidation-reduction process, pigment biosynthesis, and lipid metabolism. These DEGs from various genetic pathways suggested a complex regulatory network mediating the yellow-leaf phenotype in *Forsythia*. Since reduced chlorophyll content and abnormal chloroplast structure is observed in yellow-leaf plants, we mainly focused on the DEGs that associated with chlorophyll biosynthesis and chloroplast development. Meanwhile, the physiological experiments showed that the leaf color as well as chlorophyll content were consistently lower in yellow-leaf plants comparing to green-leaf lines given different light conditions. Therefore, we further searched for candidate gene displaying consistent expression patterns between yellow-leaf and green-leaf plants under different light intensities to narrow down the candidate gene set.

Among the chlorophyll biosynthetic genes, we observed significant down-regulation of *ChlH* in the chlorophyll-deficient yellow-leaf lines in both natural and shade conditions and considered *ChlH* for functional validation. On the other hand, we screened out *POLGAMMA2* as another candidate gene as result of its consistently down-regulation in yellow-leaf *Forsythia* lines matching the chlorophyll content and leaf color change among different groups. *POLGAMMA2* encodes a nuclear-encoded organelle DNA polymerase responsible for the replication of mitochondria and plastid genome (Parent et al., 2011). POLGAMMA2, also known as POLYMERASE I A (Pol1A), is one of the two Plant Organellar DNA polymerases (POPs) in flowering plants (García-Medel et al., 2019; Ji and Day, 2020). POLGAMMA2 is mainly involved in DNA replication, while its paralog POLGAMMA1 serves an additional role in DNA repair (Parent et al., 2011; Morley et al., 2019). In *Arabidopsis*, plants with mutation in either *POLGAMMA2* or *POLGAMMA1* can survive with no visible growth defects despite an 30% decrease in organelle DNA copy number, while deletions of both genes are lethal (Parent et al., 2011; Morley and Nielsen, 2016; Morley et al., 2019). Whether POLGAMMA2 affect chloroplast development is worthy testing in *Forsythia* species.

### Functional role of *CHLH* and *POLGAMMA2* in modulating yellow-leaf coloration

To investigate the functions of these two candidate genes, we transiently silenced *ChlH* and *POLGAMMA2* in green-leaf *Forsythia* plants and observed yellow-leaf phenotype with significant reduction of chlorophyll and carotenoid content in both *ChlH*-silenced and *POLGAMMA2*-silenced plants. We observed severely disintegrated thylakoid membranes in *FsPOLGAMMA2*-silenced plants, similar as the chloroplast structure observed in yellow-leaf *Forsythia*. While the defects of granum structures in *FsChlH*-silenced plants were less severe than that of yellow leaf *Forsythia* lines. In pea, the virus-induced gene silencing of *ChlH* also led to lower chlorophyll accumulation, undeveloped thylakoid membranes, and reduced photosynthesis in yellow-leaf mutants (Luo et al., 2013). The less disrupted chloroplasts in *FsChlH*-silenced *Forsythia* is likely dependent on the degree of *ChlH* gene down-regulation. We also validated the function of *ChlH* and *POLGAMMA2* in model plant species. The *FsChlH*-silenced tomato and tobacco plants both exhibited yellow leaves with altered chloroplast ultrastructure, which is consistent with tobacco plants with endogenous *ChlH* gene silenced (Hiriart et al., 2002). On the other hand, paled colored leaves with disassembled thylakoid layers were observed in *POLGAMMA2*-silenced plants. Previous works showed that POLYMERASE I A (Pol1A) and POLYMERASE I B (Pol1B), designated as POLGAMMA2 and POLGAMMA1, respectively, were organellar DNA polymerases known as Plant Organellar DNA Polymerases (POPs). Most flowering plants harbor two POP genes, while the dicot *Solanaceae* species, such as *Solanum lycopersicum* and *Nicotiana tomentosiformis*, contain only one POP gene (Ji and Day, 2020). It likely that the virus-induced silencing of *FsPOLGAMMA2* severely disrupted the DNA replication within chloroplasts, leading to severely disrupted chloroplast development in tobacco and tomato.

Furthermore, we detected increased chlorophyll content in the yellow-leaf plants with *ChlH* or *POLGAMMA2* transiently over-expressed. The expression of *ChlH* failed to restore the chloroplast structure, while the expression of *POLGAMMA2* partially restored the thylakoid membranes, suggesting that *POLGAMMA2* may have an additional role in chloroplast development though regulating chloroplast DNA replication. Though the transient over-expression of *ChlH* and *POLGAMMA2* caused the increment of chlorophyll content, however, the induced expression of these two genes are not sufficient for complete restoration of thylakoid membranes in yellow-leaf plants. It is likely due to the limited efficacy of transient assays that may remain active within only a few days (Wroblewski et al., 2005). Another putative explanation is that there are other casual genes participating in the formation of yellow-leaf *Forsythia*. In addition to genes directly involved in pigment synthesis and chloroplast structure, there are other genetic factors influencing the leaf color through mediating processes such as chloroplast gene transcription and translation, plastid-nuclear signaling, and ROS scavenging system (Pogson and Albrecht, 2011; Cheng et al., 2022). With comparative transcriptome sequencing approach, we are only able to detect functional candidate genes with significant fold-change between yellow-leaf and green-leaf lines. The underlying genetic mechanism of yellow-leaf coloration may still remain incomplete. In the future studies, genetic mapping approaches can be applied to further narrow down the candidate gene list and elucidate the casual mechanism of yellow-leaf coloration in *Forsythia*.

To further understand the functional mechanism of *ChlH* and *POLGAMMA2*, we performed RNA-seq on the green-leaf *Forsythia* plants with *ChlH* and *POLGAMMA2* silenced, respectively. The silencing of *ChlH* gene leads to decreased abundance of a large number of genes that involved in chlorophyll and carotenoid biosynthesis, photosynthesis, and chloroplast development. *ChlH* is a multifunctional protein coordinating the chlorophyll biosynthesis and plastid-to-nucleus retrograde signaling (Ibata et al., 2016). In *Arabidopsis*, the *ChlH* mutant displayed repressed plastid signaling with decreased level of photosynthesis-associated nuclear genes, such as *LHCB1* (Schlicke et al., 2014). Since chlorophyll biosynthesis is tightly coupled with thylakoid membrane development, the chlorophyll deficiency triggered by *ChlH* silencing may indirectly impair the transcription of genes regulating chloroplast biogenesis and thylakoid membranes assembly, therefore leading to undeveloped chloroplast structure in *Forsythia* (Hiriart et al., 2002).

POLGAMMA2 is an organelle localized DNA polymerase associated with DNA replication in chloroplast (Morley and Nielsen, 2016). The modified transcription level of *POLGAMMA2* can directly affect the chloroplast DNA copy number and influence chloroplast development related genes indirectly. When *POLGAMMA2* is transiently silenced in *Forsythia*, we observed strong reduction of chlorophyll and carotenoid synthetic genes, which correlates with the deficiency in chlorophyll and carotenoid. We also observed similar yellow-leaf phenotype in tomato and tobacco with *POLGAMMA2* transiently silenced, indicating the functional conservation of *POLGAMMA2*. Moreover, a few genes that possibly regulating thylakoidal membrane formation and architecture were also found repressed after the silencing of *POLGAMMA2*. *CURT1* is an essential gene localized to grana margin and is required for thylakoid membrane bending and the granum stacking (Armbruster et al., 2013). In Arabidopsis, the thylakoids are disorganized with unstacked membranes in the *CURT1* mutants (Armbruster et al., 2013). *THF1* encodes an imported chloroplast protein that regulate the thylakoid membrane biogenesis and organization during early stage of chloroplast development (Wang et al., 2004). The loss function in *THF1* lead to reduced chlorophyll level, less efficient photosynthesis, and defected thylakoid stacking, resulting in variegated leaves in *Arabidopsis* (Wang et al., 2004). In *Forsythia*, the silencing of *POLGAMMA2* may somehow disrupt the photosynthetic pigment accumulation and thylakoid membrane organization through repressing the transcription of chlorophyll synthesis genes, such as *ChlH* and *PORA*, and chloroplast development related genes, such as *THF1* and *CURT1*. Taken together, the inhibition of *ChlH* and *POLGAMMA2* is likely required for the yellow-leaf *Forsythia* through influencing the transcription of genes related to chlorophyll biosynthesis and chloroplast structure intactness. In the current study, we only characterized the functional role of candidate DEGs with consistent expression pattern regardless of light-intensity change. Future research can focus on the candidate genes responsive to light-intensity change and test their functional roles in regulating pigment synthesis and thylakoid structure in *Forsythia*.

## Conclusion

To conclude, our study characterized the yellow-leaf *Forsythia* with reduced chlorophyll and carotenoid content, as well as defected chloroplast structure. The transcriptome sequencing and expression pattern analysis screened out two candidate genes, *ChlH* and *POLGAMMA2*, with expression profiles strongly correlated with chlorophyll content and leaf color variation among different leaf-colored *Forsythia* lines. The transient silencing of *ChlH* and *POLGAMMA2* lead to reduced chlorophyll level and disrupted chloroplast structure, while the transient over-expression of these two genes promoted the restoration of thylakoid architecture in yellow-leaf *Forsythia*. By examining genes with transcriptional change responsive to the silencing of these two genes, we conclude that *ChlH* and *POLGAMMA2* may be related to the yellow-leaf phenotype in *Forsythia* through directly or indirectly mediating genes involved in chlorophyll biosynthesis and chloroplast development. In general, our study provides a deep understanding in the physiological as well as molecular mechanism underlying the yellow-leaf coloration in *Forsythia*.

## Data availability statement

The datasets presented in this study can be found in online repositories. The names of the repository/repositories and accession number(s) can be found in the article/Supplementary material.

## Author contributions

HP conceived the experiments. MZ, JS, and YW performed the experiments and wrote the manuscript. ZZ, XZ, TC, JW, and QZ assisted in analyzing the data and finalizing the manuscript. All authors contributed to the article and approved the submitted version.
