# Supplementary material for: Comparative transcriptome analysis identified ChlH and POLGAMMA2 in regulating yellow-leaf coloration in Forsythia
Source: Front Plant Sci. 2022 Sep 9;13:1009575. doi: 10.3389/fpls.2022.1009575 (PMC9501713; doi:10.3389/fpls.2022.1009575)
Supplement: Supplementary file 1 [file Data_Sheet_1.DOCX]

Supplementary Material

# Supplementary Figures

**Figure S1. The functional enrichment analysis of DEGs between yellow-leaf and green-leaf *Forsythia* plants.**

(**A**) The GO terms enriched among DEGs between group L1 vs L2; (**B**) The GO terms enriched among DEGs between group S1 vs S2.

**Figure S2. The qPCR validation of DEGs detected with RNA-seq analysis.**


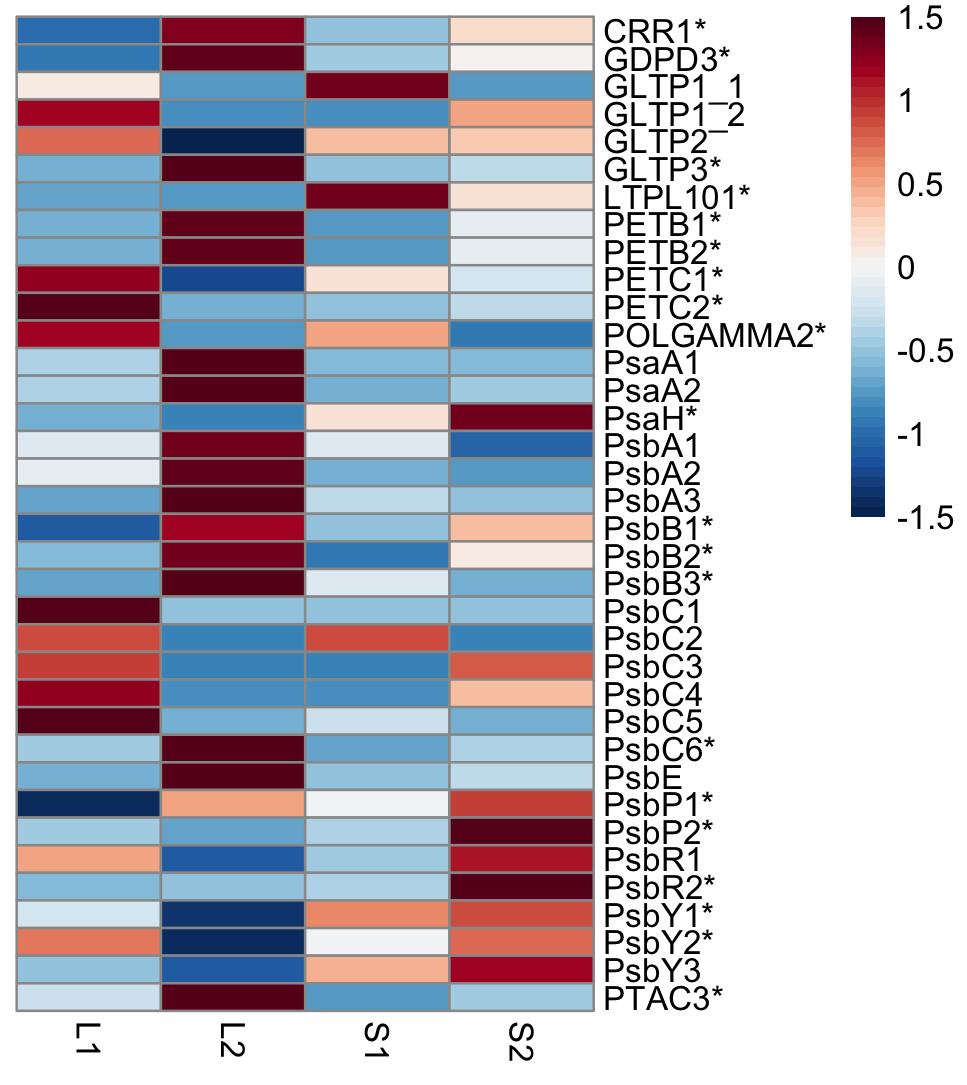


**Figure S3. Expression pattern of DEGs putatively involved in photosynthesis and chloroplast development.**


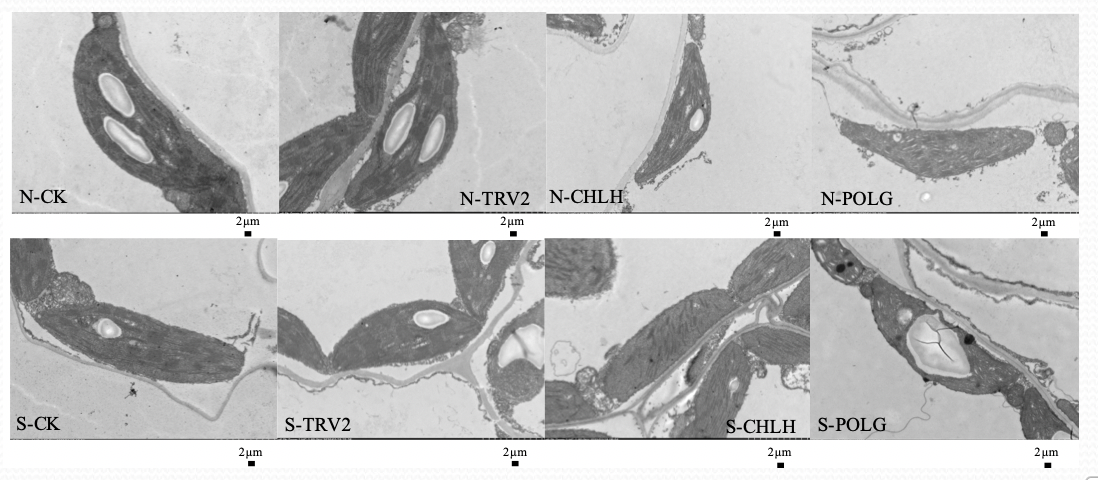


**Figure S4. The chloroplast ultrastructure of noninfected plants (N/S-CK), vector controls (N/S-TRV2), *ChlH*-silenced (N/S-CHLH), and *POLGAMMA2*-silenced (N/S-POLG) tobacco (N-) and tomato (S-).**


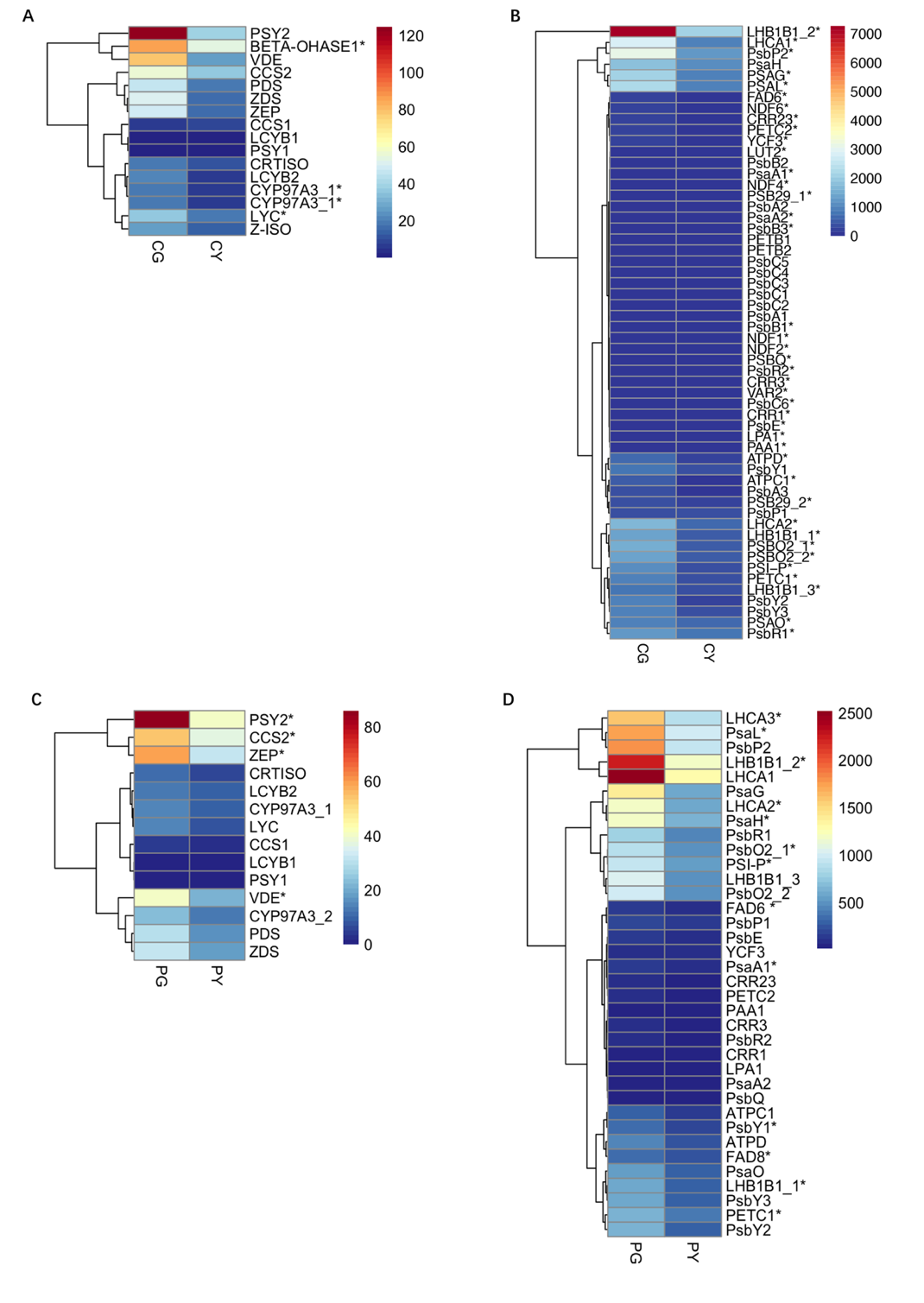


**Figure S5. Expression pattern of DEGs involved in carotenoid biosynthesis and photosynthesis between CG and CY (A-B), and between PG and PY (C-D).**
