# Supplementary material for: Comparative transcriptome analysis identified ChlH and POLGAMMA2 in regulating yellow-leaf coloration in Forsythia
Source: Front Plant Sci. 2022 Sep 9;13:1009575. doi: 10.3389/fpls.2022.1009575 (PMC9501713; doi:10.3389/fpls.2022.1009575)
Supplement: Supplementary file 2 [file Data_Sheet_2.docx]

Supplementary Material

# Supplementary Tables

**Table S1. Primers of selected DEGs used in the qPCR assays.**

| **Name** | **Forward primers** | **Reverse primers** | **Category** |
| --- | --- | --- | --- |
| F-*ChlH* | GGTGAAAATCGTGGAGGAAA | ATGTCGCCTCTCAATCCATC | gene expression detection |
| F-*POLGAMMA2* | ACCAGCGATCAACTGTTTCC | TGTGCTTCTTGAGTGGCATC |  |
| F-*ChlE* | TCCTAACGAAGGCCAGAAAA | ATTCTCGTCTTGGCACCAGT |  |
| F-*PAO* | GGCTCGGTACAACAAGAAGC | GCCAAAAGAACAACCCAGAA |  |
| F-*CAO1* | TTGCCAGGTTTTGAGATTCC | GTGATGGAACTCCCCGTAGA |  |
| F-*HY2* | ATCCAGGCCATCAAGTTTTG | TTTTCGCCAATGAATTCTCC |  |
| F-*PSY* | TGACACCTTTCTCTGCATCG | GATTGCAAATCAGCTGACGA |  |
| F-*CRTISO* | TGGGAGTTCAGGGTTTTACG | ACTAGGACCGAGAGGCCATT |  |
| F-*FNR1* | GTCTCTGTTGAGAGGCTTGTT | CCAGGAGGAATGATCCCATAAC |  |
| F-*CYTC6A* | CTCGCCGCAGAAGATAGATAAA | GCAGAGAGGAGATAATGCTACAA |  |
| F-*PETC1* | GCTGGTTCCTTATGCTTCTTTC | CATTCCCAAGGGCATCCTTA |  |
| F-GGP | GCTCCTCGACACTCAAGTTAAT | CCTCCTCATAGTCCTTCTTCCT |  |
| F-*GAL1_2* | GCACTCGGACTTGATTCTTCT | TAAAGGAGCCCACGCTATTG |  |
| F-*GAL2_1* | CTGCTGCCTGTTCATCCTTATT | GGTGTATCAGCCAACCCATTAC |  |
| F-*CM1* | GTAGCGATGGTGAAGAGCTATC | GATCGGAGCACAGTGTTTCT |  |
| F-*Cyclophilin* | CCGGAATGGATGTGGTGTATAA | GGGAAGTTCACCACTGTCTG |  |
| F-ChlH | GCTGCTTCGGGATGGAATA | CAGTTCTCCCTCTTCTGGATTT | gene silencing detection in *Forsythia* |
| F-POLGAMMA2 | TAAAGGAGCCGGAGGCTTAT | TGTATCCGCACATTCGTGAAG |  |
| S-*ChlH* | ACTTTCTACGACCCAAGATT | CCTCTTTGGCTTCTTGATTAC | gene silencing detection in tomato |
| S- *POLGAMMA2* | AGTCTCTGGTTCAATTCCAAG | ACCTGCTACCACCCATAA |  |
| N-*ChlH* | ACAATGGCTTCTTTGGTTTC | GGGAAGAAATGAGTGAAGAAAG | gene silencing detection in tobacco |
| N- *POLGAMMA2* | TTCTGCCACAACCTGTATTT | CTCCAGTTTCCAACTACCTTC |  |
| TRV2 | TGTTTGAGGGAAAAGTAGAGAACGT | TTACCGATCAATCAAGATCAGTCGA | gene expression detection |
| N-Actin | CCTGAGGTCCTTTTCCAACCA | GGATTCCGGCAGCTTCCATT | gene silencing detection in tobacco |
| S-Actin | GGAATGGGACAGAAGGAT | CAGTCAGGAGAACAGGGT | gene silencing detection in tomato |

**Table S2. Primers used for plasmid construction in the study.**

| **Name** | **Forward primers** | **Reverse primers** | **Category** |
| --- | --- | --- | --- |
| SS-*ChlH* | ATGCTGGTTGATCGTGCCTATCT | GGCTGAATACCAGTTCTGCGCACATTT | Full-length *Forsythia* gene CDS cloning |
| SS- *POLGAMMA2* | ATGCTGGTTGATCGTGCCTATC | CTTGGCTGAATACCAGTTCTGCGC |  |
| F-*ChlH*-VIGS | TAAGGTTACCGAATTCTCTACTAAATGGTCATCC | GCTCGGTACCGGATCCGATAGAGGAACAGGGAAGT | gene fragment cloning for VIGS |
| F-*POLGAMMA2*-VIGS | TAAGGTTACCGAATTCGCCTCCATCCAAGCTCC | GCTCGGTACCGGATCCTCTGAAAGACGAAAAGCGAA |  |
| S-*ChlH*-VIGS | TAAGGTTACCGAATTTCTGAGCCTGTTCTT | GCTCGGTACCGGATCAAAGACACAAACACA |  |
| S-*POLGAMMA2*-VIGS | TAAGGTTACCGAATTGCCGCTGAAATCTTT | GCTCGGTACCGGATCGTCTTTCTGTTCAATCT |  |
| N-*ChlH*-VIGS | TAAGGTTACCGAATTCCTTGGGAAGAAATGAGTG | GCTCGGTACCGGATCCACTACTCTCCACATAAAAC |  |
| N-*POLGAMMA2*-VIGS | TAAGGTTACCGAATTCTCTCTCCAGTTTCCAAC | GCTCGGTACCGGATCCAACTCAGAGTGTAGA |  |

**Table S3. The leaf anatomical features of green-leaf and yellow-leaf plants under different light intensities.**

| Group | Palisade thickness (μm) | Spongy thickness (μm) | Leaf thickness (μm) | Epidermis thickness (μm) | SR (%) | CTR (%) |
| --- | --- | --- | --- | --- | --- | --- |
| L1 | 82±9 a | 122±4 b | 238±9 a | 3.61±0.31 a | 52±4 b | 35±2 a |
| L2 | 36±4 c | 150±4 a | 226±9 a | 2.44±0.23 b | 66±3 a | 16±1 c |
| S1 | 56±3 b | 107±8 c | 196±2 b | 4.07±0.36 a | 54±4 b | 29±4 b |
| S2 | 27±2 c | 98±3 c | 155±2 c | 1.98±0.16 b | 63±2 a | 17±1 c |

CTR= Palisade thickness/ Leaf thickness; SR= Spongy thickness/ Leaf thickness. For each parameter, Mean±SE followed with the different alphabetic letter are significantly different in Duncan’s Multiple Range test under significance level 0.05.

**Table S4. The analysis of chloroplast structure in green-leaf and yellow-leaf plants under different light intensities.**

| Group | Chloroplast length (μm) | Chloroplast width (μm) | Number of chloroplasts per cell | Thylakoid thickness (μm) | Thylakoids per chloroplast | Number of starch granules per chloroplast |
| --- | --- | --- | --- | --- | --- | --- |
| L1 | 3.6±0.2 c | 1.7±0.3 a | 7.4±0.5 a | 0.2±0.0 a | 9.2±1.3 a | 0.8±0.4 a |
| L2 | 4.8±1.0 ab | 1.8±0.1 a | 8.6±0.9 a | / | / | 1.2±0.4 a |
| S1 | 5.3±1.1 a | 1.9±0.6 a | 8.4±1.5 a | 0.3±0.1 a | 10.8±2.3 a | 1.2±0.4 a |
| S2 | 3.9±0.4 bc | 1.6±0.3 a | 7.8±0.8 a | 0.2±0.1 a | 9.2±1.5 a | 0.0±0.0 b |

For each parameter, Mean±SE followed with the different alphabetic letter are significantly different in Duncan’s Multiple Range test under significance level 0.05.

**Table S5. RNA-seq reads generated from leaf samples of four groups.**

| Sample | Total Raw Reads (M) | Total Clean Reads (M) | Total Clean Bases (Gb) | Clean Reads Q20 (%) | Clean Reads Q30 (%) | Clean Reads Ratio (%) | Total Mapping (%) |
| --- | --- | --- | --- | --- | --- | --- | --- |
| L1_1 | 72.99 | 71.26 | 10.64 | 97.01 | 92.35 | 97.64 | 77.21 |
| L1_2 | 61.52 | 59.82 | 8.94 | 96.84 | 92 | 97.23 | 78.33 |
| L1_3 | 76.76 | 74.56 | 11.14 | 96.83 | 91.97 | 97.14 | 78.36 |
| L2_1 | 62.7 | 61.77 | 9.24 | 96.84 | 91.94 | 98.52 | 76.97 |
| L2_2 | 67.2 | 66.12 | 9.89 | 96.74 | 91.78 | 98.4 | 76.54 |
| L2_3 | 62.81 | 61.91 | 9.26 | 96.82 | 91.92 | 98.57 | 75.65 |
| S1_1 | 69.69 | 67.7 | 10.1 | 96.67 | 91.64 | 97.16 | 77.35 |
| S1_2 | 66.84 | 65.26 | 9.74 | 96.81 | 91.9 | 97.63 | 78.02 |
| S1_3 | 64.54 | 62.76 | 9.38 | 96.65 | 91.5 | 97.24 | 77.5 |
| S2_1 | 73.51 | 71.68 | 10.71 | 96.86 | 92.07 | 97.5 | 76.95 |
| S2_2 | 71.48 | 69.57 | 10.39 | 96.89 | 92.2 | 97.32 | 76.52 |
| S2_3 | 74.37 | 72.18 | 10.79 | 96.79 | 91.99 | 97.06 | 76.16 |

**Table S6. The GO terms of biological processes enriched among DEGs that detected in group comparison L1 vs L2 and S1 vs S2.**

| Comparison | GO term | Description | P value | FDR |
| --- | --- | --- | --- | --- |
| L1_vs_L2 | GO:0008152 | metabolic process | 2.46E-51 | 1.56E-48 |
| L1_vs_L2 | GO:0044699 | single-organism process | 1.19E-19 | 3.78E-17 |
| L1_vs_L2 | GO:0044710 | single-organism metabolic process | 3.81E-17 | 9.68E-15 |
| L1_vs_L2 | GO:0055114 | oxidation-reduction process | 1.24E-12 | 2.25E-10 |
| L1_vs_L2 | GO:0071704 | organic substance metabolic process | 3.89E-11 | 6.18E-09 |
| L1_vs_L2 | GO:0006259 | DNA metabolic process | 1.64E-10 | 2.31E-08 |
| L1_vs_L2 | GO:0044238 | primary metabolic process | 2.46E-09 | 2.61E-07 |
| L1_vs_L2 | GO:0005975 | carbohydrate metabolic process | 3.72E-08 | 3.38E-06 |
| L1_vs_L2 | GO:0043043 | peptide biosynthetic process | 4.32E-07 | 3.23E-05 |
| L1_vs_L2 | GO:0006412 | translation | 5.35E-07 | 3.69E-05 |
| L1_vs_L2 | GO:0006996 | organelle organization | 5.81E-07 | 3.69E-05 |
| L1_vs_L2 | GO:0006518 | peptide metabolic process | 7.18E-07 | 3.97E-05 |
| L1_vs_L2 | GO:0043604 | amide biosynthetic process | 7.48E-07 | 3.97E-05 |
| L1_vs_L2 | GO:0043603 | cellular amide metabolic process | 8.34E-07 | 4.24E-05 |
| L1_vs_L2 | GO:0005992 | trehalose biosynthetic process | 2.93E-06 | 1.15E-04 |
| L1_vs_L2 | GO:0044723 | single-organism carbohydrate metabolic process | 2.98E-06 | 1.15E-04 |
| L1_vs_L2 | GO:0005991 | trehalose metabolic process | 4.27E-06 | 1.47E-04 |
| L1_vs_L2 | GO:0046351 | disaccharide biosynthetic process | 6.10E-06 | 1.99E-04 |
| L1_vs_L2 | GO:0009987 | cellular process | 6.60E-06 | 2.10E-04 |
| L1_vs_L2 | GO:0051276 | chromosome organization | 1.06E-05 | 3.07E-04 |
| L1_vs_L2 | GO:0019725 | cellular homeostasis | 1.41E-05 | 3.58E-04 |
| L1_vs_L2 | GO:0045454 | cell redox homeostasis | 1.43E-05 | 3.58E-04 |
| L1_vs_L2 | GO:0034613 | cellular protein localization | 1.44E-05 | 3.58E-04 |
| L1_vs_L2 | GO:0070727 | cellular macromolecule localization | 1.44E-05 | 3.58E-04 |
| L1_vs_L2 | GO:0044262 | cellular carbohydrate metabolic process | 1.77E-05 | 4.20E-04 |
| L1_vs_L2 | GO:0006396 | RNA processing | 1.78E-05 | 4.20E-04 |
| L1_vs_L2 | GO:0005984 | disaccharide metabolic process | 1.78E-05 | 4.20E-04 |
| L1_vs_L2 | GO:0008104 | protein localization | 2.33E-05 | 5.29E-04 |
| L1_vs_L2 | GO:0006281 | DNA repair | 2.79E-05 | 6.11E-04 |
| L1_vs_L2 | GO:0009312 | oligosaccharide biosynthetic process | 2.87E-05 | 6.20E-04 |
| L1_vs_L2 | GO:0034637 | cellular carbohydrate biosynthetic process | 3.23E-05 | 6.74E-04 |
| L1_vs_L2 | GO:0006886 | intracellular protein transport | 3.76E-05 | 7.59E-04 |
| L1_vs_L2 | GO:0045184 | establishment of protein localization | 3.98E-05 | 7.78E-04 |
| L1_vs_L2 | GO:0016051 | carbohydrate biosynthetic process | 4.79E-05 | 9.08E-04 |
| L1_vs_L2 | GO:0015031 | protein transport | 5.47E-05 | 9.94E-04 |
| L1_vs_L2 | GO:0015833 | peptide transport | 5.47E-05 | 9.94E-04 |
| L1_vs_L2 | GO:0033036 | macromolecule localization | 5.65E-05 | 1.01E-03 |
| L1_vs_L2 | GO:0071840 | cellular component organization or biogenesis | 6.53E-05 | 1.12E-03 |
| L1_vs_L2 | GO:1901566 | organonitrogen compound biosynthetic process | 6.76E-05 | 1.15E-03 |
| L1_vs_L2 | GO:0050794 | regulation of cellular process | 7.09E-05 | 1.19E-03 |
| L1_vs_L2 | GO:0009311 | oligosaccharide metabolic process | 7.29E-05 | 1.20E-03 |
| L1_vs_L2 | GO:0016192 | vesicle-mediated transport | 8.19E-05 | 1.31E-03 |
| L1_vs_L2 | GO:0033554 | cellular response to stress | 8.21E-05 | 1.31E-03 |
| L1_vs_L2 | GO:0006974 | cellular response to DNA damage stimulus | 1.35E-04 | 1.98E-03 |
| L1_vs_L2 | GO:0050789 | regulation of biological process | 1.53E-04 | 2.22E-03 |
| L1_vs_L2 | GO:0051716 | cellular response to stimulus | 1.94E-04 | 2.75E-03 |
| L1_vs_L2 | GO:0055085 | transmembrane transport | 2.17E-04 | 3.03E-03 |
| L1_vs_L2 | GO:0044763 | single-organism cellular process | 2.54E-04 | 3.35E-03 |
| L1_vs_L2 | GO:0005977 | glycogen metabolic process | 2.58E-04 | 3.35E-03 |
| L1_vs_L2 | GO:0005978 | glycogen biosynthetic process | 2.58E-04 | 3.35E-03 |
| L1_vs_L2 | GO:0006112 | energy reserve metabolic process | 2.58E-04 | 3.35E-03 |
| L1_vs_L2 | GO:0044267 | cellular protein metabolic process | 3.35E-04 | 4.26E-03 |
| L1_vs_L2 | GO:0051641 | cellular localization | 3.78E-04 | 4.72E-03 |
| L1_vs_L2 | GO:0065007 | biological regulation | 4.46E-04 | 5.45E-03 |
| L1_vs_L2 | GO:0009073 | aromatic amino acid family biosynthetic process | 4.74E-04 | 5.69E-03 |
| L1_vs_L2 | GO:0042886 | amide transport | 6.85E-04 | 7.71E-03 |
| L1_vs_L2 | GO:0051649 | establishment of localization in cell | 7.84E-04 | 8.75E-03 |
| L1_vs_L2 | GO:0046907 | intracellular transport | 8.58E-04 | 9.33E-03 |
| L1_vs_L2 | GO:0016043 | cellular component organization | 9.17E-04 | 9.72E-03 |
| L1_vs_L2 | GO:0034641 | cellular nitrogen compound metabolic process | 9.73E-04 | 1.02E-02 |
| L1_vs_L2 | GO:0006662 | glycerol ether metabolic process | 1.06E-03 | 1.08E-02 |
| L1_vs_L2 | GO:0018904 | ether metabolic process | 1.06E-03 | 1.08E-02 |
| L1_vs_L2 | GO:0006355 | regulation of transcription, DNA-templated | 1.07E-03 | 1.08E-02 |
| L1_vs_L2 | GO:1903506 | regulation of nucleic acid-templated transcription | 1.07E-03 | 1.08E-02 |
| L1_vs_L2 | GO:2001141 | regulation of RNA biosynthetic process | 1.07E-03 | 1.08E-02 |
| L1_vs_L2 | GO:0019438 | aromatic compound biosynthetic process | 1.09E-03 | 1.08E-02 |
| L1_vs_L2 | GO:0051252 | regulation of RNA metabolic process | 1.09E-03 | 1.08E-02 |
| L1_vs_L2 | GO:0019219 | regulation of nucleobase-containing compound metabolic process | 1.31E-03 | 1.27E-02 |
| L1_vs_L2 | GO:0006351 | transcription, DNA-templated | 1.43E-03 | 1.35E-02 |
| L1_vs_L2 | GO:0097659 | nucleic acid-templated transcription | 1.43E-03 | 1.35E-02 |
| L1_vs_L2 | GO:0010556 | regulation of macromolecule biosynthetic process | 1.44E-03 | 1.35E-02 |
| L1_vs_L2 | GO:2000112 | regulation of cellular macromolecule biosynthetic process | 1.44E-03 | 1.35E-02 |
| L1_vs_L2 | GO:0009889 | regulation of biosynthetic process | 1.47E-03 | 1.35E-02 |
| L1_vs_L2 | GO:0031326 | regulation of cellular biosynthetic process | 1.47E-03 | 1.35E-02 |
| L1_vs_L2 | GO:0032774 | RNA biosynthetic process | 1.48E-03 | 1.35E-02 |
| L1_vs_L2 | GO:0010468 | regulation of gene expression | 1.73E-03 | 1.55E-02 |
| L1_vs_L2 | GO:1901362 | organic cyclic compound biosynthetic process | 1.78E-03 | 1.58E-02 |
| L1_vs_L2 | GO:0051171 | regulation of nitrogen compound metabolic process | 1.82E-03 | 1.61E-02 |
| L1_vs_L2 | GO:0080090 | regulation of primary metabolic process | 1.96E-03 | 1.72E-02 |
| L1_vs_L2 | GO:0006952 | defense response | 2.02E-03 | 1.76E-02 |
| L1_vs_L2 | GO:0044281 | small molecule metabolic process | 2.16E-03 | 1.83E-02 |
| L1_vs_L2 | GO:0031323 | regulation of cellular metabolic process | 2.17E-03 | 1.83E-02 |
| L1_vs_L2 | GO:0044085 | cellular component biogenesis | 2.20E-03 | 1.83E-02 |
| L1_vs_L2 | GO:1901616 | organic hydroxy compound catabolic process | 2.26E-03 | 1.83E-02 |
| L1_vs_L2 | GO:0046164 | alcohol catabolic process | 2.26E-03 | 1.83E-02 |
| L1_vs_L2 | GO:0046174 | polyol catabolic process | 2.26E-03 | 1.83E-02 |
| L1_vs_L2 | GO:0019310 | inositol catabolic process | 2.26E-03 | 1.83E-02 |
| L1_vs_L2 | GO:0060255 | regulation of macromolecule metabolic process | 2.28E-03 | 1.83E-02 |
| L1_vs_L2 | GO:0071555 | cell wall organization | 2.62E-03 | 2.07E-02 |
| L1_vs_L2 | GO:0042592 | homeostatic process | 2.68E-03 | 2.10E-02 |
| L1_vs_L2 | GO:0034654 | nucleobase-containing compound biosynthetic process | 2.76E-03 | 2.14E-02 |
| L1_vs_L2 | GO:0010033 | response to organic substance | 2.76E-03 | 2.14E-02 |
| L1_vs_L2 | GO:0019222 | regulation of metabolic process | 2.83E-03 | 2.18E-02 |
| L1_vs_L2 | GO:0045229 | external encapsulating structure organization | 2.85E-03 | 2.19E-02 |
| L1_vs_L2 | GO:0034660 | ncRNA metabolic process | 3.22E-03 | 2.43E-02 |
| L1_vs_L2 | GO:0009719 | response to endogenous stimulus | 3.76E-03 | 2.75E-02 |
| L1_vs_L2 | GO:0009725 | response to hormone | 3.76E-03 | 2.75E-02 |
| L1_vs_L2 | GO:0009072 | aromatic amino acid family metabolic process | 3.82E-03 | 2.78E-02 |
| L1_vs_L2 | GO:0006810 | transport | 3.85E-03 | 2.78E-02 |
| L1_vs_L2 | GO:0051179 | localization | 4.11E-03 | 2.92E-02 |
| L1_vs_L2 | GO:0044275 | cellular carbohydrate catabolic process | 4.24E-03 | 3.00E-02 |
| L1_vs_L2 | GO:0051234 | establishment of localization | 4.29E-03 | 3.02E-02 |
| L1_vs_L2 | GO:0044237 | cellular metabolic process | 4.41E-03 | 3.08E-02 |
| L1_vs_L2 | GO:0044282 | small molecule catabolic process | 4.47E-03 | 3.11E-02 |
| L1_vs_L2 | GO:0065008 | regulation of biological quality | 5.44E-03 | 3.64E-02 |
| L1_vs_L2 | GO:0008652 | cellular amino acid biosynthetic process | 5.87E-03 | 3.91E-02 |
| L1_vs_L2 | GO:0018130 | heterocycle biosynthetic process | 6.13E-03 | 4.06E-02 |
| L1_vs_L2 | GO:0009058 | biosynthetic process | 6.26E-03 | 4.13E-02 |
| L1_vs_L2 | GO:0044706 | multi-multicellular organism process | 6.40E-03 | 4.17E-02 |
| L1_vs_L2 | GO:0009856 | pollination | 6.40E-03 | 4.17E-02 |
| L1_vs_L2 | GO:0009607 | response to biotic stimulus | 6.45E-03 | 4.18E-02 |
| L1_vs_L2 | GO:0019538 | protein metabolic process | 6.68E-03 | 4.31E-02 |
| L1_vs_L2 | GO:0006520 | cellular amino acid metabolic process | 6.71E-03 | 4.31E-02 |
| L1_vs_L2 | GO:0006020 | inositol metabolic process | 6.95E-03 | 4.42E-02 |
| L1_vs_L2 | GO:0043933 | macromolecular complex subunit organization | 7.37E-03 | 4.62E-02 |
| L1_vs_L2 | GO:0071702 | organic substance transport | 7.44E-03 | 4.64E-02 |
| L1_vs_L2 | GO:0071554 | cell wall organization or biogenesis | 7.49E-03 | 4.65E-02 |
| L1_vs_L2 | GO:1901576 | organic substance biosynthetic process | 7.55E-03 | 4.66E-02 |
| L1_vs_L2 | GO:0009664 | plant-type cell wall organization | 7.86E-03 | 4.81E-02 |
| L1_vs_L2 | GO:0009308 | amine metabolic process | 7.86E-03 | 4.81E-02 |
| L1_vs_L2 | GO:0044249 | cellular biosynthetic process | 8.15E-03 | 4.96E-02 |
| L1_vs_L2 | GO:1901605 | alpha-amino acid metabolic process | 8.19E-03 | 4.96E-02 |
| S1_vs_S2 | GO:0008152 | metabolic process | 5.48E-22 | 2.70E-19 |
| S1_vs_S2 | GO:0055114 | oxidation-reduction process | 3.68E-06 | 2.59E-04 |
| S1_vs_S2 | GO:0044710 | single-organism metabolic process | 5.03E-05 | 1.18E-03 |
| S1_vs_S2 | GO:0006355 | regulation of transcription, DNA-templated | 6.82E-05 | 1.29E-03 |
| S1_vs_S2 | GO:1903506 | regulation of nucleic acid-templated transcription | 6.82E-05 | 1.29E-03 |
| S1_vs_S2 | GO:2001141 | regulation of RNA biosynthetic process | 6.82E-05 | 1.29E-03 |
| S1_vs_S2 | GO:0051252 | regulation of RNA metabolic process | 6.91E-05 | 1.29E-03 |
| S1_vs_S2 | GO:0019219 | regulation of nucleobase-containing compound metabolic process | 7.77E-05 | 1.29E-03 |
| S1_vs_S2 | GO:0010556 | regulation of macromolecule biosynthetic process | 8.28E-05 | 1.29E-03 |
| S1_vs_S2 | GO:2000112 | regulation of cellular macromolecule biosynthetic process | 8.28E-05 | 1.29E-03 |
| S1_vs_S2 | GO:0009889 | regulation of biosynthetic process | 8.39E-05 | 1.29E-03 |
| S1_vs_S2 | GO:0031326 | regulation of cellular biosynthetic process | 8.39E-05 | 1.29E-03 |
| S1_vs_S2 | GO:0044238 | primary metabolic process | 1.01E-04 | 1.47E-03 |
| S1_vs_S2 | GO:0010468 | regulation of gene expression | 1.15E-04 | 1.62E-03 |
| S1_vs_S2 | GO:0071704 | organic substance metabolic process | 1.43E-04 | 1.87E-03 |
| S1_vs_S2 | GO:0051171 | regulation of nitrogen compound metabolic process | 1.44E-04 | 1.87E-03 |
| S1_vs_S2 | GO:0080090 | regulation of primary metabolic process | 1.51E-04 | 1.91E-03 |
| S1_vs_S2 | GO:0044699 | single-organism process | 1.62E-04 | 1.95E-03 |
| S1_vs_S2 | GO:0031323 | regulation of cellular metabolic process | 1.63E-04 | 1.95E-03 |
| S1_vs_S2 | GO:0060255 | regulation of macromolecule metabolic process | 2.02E-04 | 2.32E-03 |
| S1_vs_S2 | GO:0019222 | regulation of metabolic process | 2.34E-04 | 2.63E-03 |
| S1_vs_S2 | GO:0006351 | transcription, DNA-templated | 5.18E-04 | 5.31E-03 |
| S1_vs_S2 | GO:0097659 | nucleic acid-templated transcription | 5.18E-04 | 5.31E-03 |
| S1_vs_S2 | GO:0032774 | RNA biosynthetic process | 5.28E-04 | 5.31E-03 |
| S1_vs_S2 | GO:0050794 | regulation of cellular process | 1.08E-03 | 9.47E-03 |
| S1_vs_S2 | GO:1901566 | organonitrogen compound biosynthetic process | 1.11E-03 | 9.59E-03 |
| S1_vs_S2 | GO:0034654 | nucleobase-containing compound biosynthetic process | 1.38E-03 | 1.14E-02 |
| S1_vs_S2 | GO:0006012 | galactose metabolic process | 1.61E-03 | 1.30E-02 |
| S1_vs_S2 | GO:0050789 | regulation of biological process | 1.84E-03 | 1.44E-02 |
| S1_vs_S2 | GO:0018130 | heterocycle biosynthetic process | 2.53E-03 | 1.88E-02 |
| S1_vs_S2 | GO:0019438 | aromatic compound biosynthetic process | 2.55E-03 | 1.88E-02 |
| S1_vs_S2 | GO:0016070 | RNA metabolic process | 2.98E-03 | 2.07E-02 |
| S1_vs_S2 | GO:1901362 | organic cyclic compound biosynthetic process | 3.18E-03 | 2.17E-02 |
| S1_vs_S2 | GO:0065007 | biological regulation | 4.24E-03 | 2.78E-02 |
| S1_vs_S2 | GO:0005975 | carbohydrate metabolic process | 5.59E-03 | 3.63E-02 |
| S1_vs_S2 | GO:0006357 | regulation of transcription from RNA polymerase II promoter | 7.56E-03 | 4.60E-02 |

**Table S7. The DEGs involved in chlorophyll and carotenoid biosynthesis and metabolism.**

| Pathway | Name | Description | ID | Qvalue_L1vsL2 | Qvalue_S1vsS2 |
| --- | --- | --- | --- | --- | --- |
| Carotenoid biosynthesis | CRTISO | Flavin containing amine oxidoreductase | EVM0007104 | 1.15E-01 | 9.81E-01 |
| Carotenoid biosynthesis | PSY2 | Squalene/phytoene synthase | EVM0028954 | 9.98E-09 | 2.27E-01 |
| Carotenoid biosynthesis | ZDS | Flavin containing amine oxidoreductase | EVM0001873 | 3.10E-03 | 5.86E-01 |
| Carotenoid biosynthesis | ZEP | FAD binding domain | EVM0031387 | 1.59E-01 | 2.50E-03 |
| Chlorophyll biosynthesis | CAO1 | Pheophorbide a oxygenase | EVM0015069 | 3.42E-01 | 6.87E-03 |
| Chlorophyll biosynthesis | CAO2 | Rieske [2Fe-2S] domain | EVM0020970 | 9.61E-05 | 1.78E-03 |
| Chlorophyll biosynthesis | CHL I | Magnesium chelatase, subunit I | EVM0014337 | 3.70E-02 | 1.02E-06 |
| Chlorophyll biosynthesis | CHLH | Magnesium chelatase, subunit H | EVM0022314 | 4.44E-03 | 8.61E-02 |
| Chlorophyll biosynthesis | CHLM | Magnesium-protoporphyrin IX methyltransferase C-terminus | EVM0011329 | 2.18E-10 | 3.10E-03 |
| Chlorophyll biosynthesis | CLH | Chlorophyllase | EVM0024022 | 6.90E-01 | 9.37E-01 |
| Chlorophyll biosynthesis | CRD1 | Rubrerythrin | EVM0007348 | 3.16E-04 | 5.28E-02 |
| Chlorophyll biosynthesis | CRD2 | Rubrerythrin | EVM0025856 | 9.88E-05 | 2.02E-05 |
| Chlorophyll biosynthesis | DVR | NAD(P)H-binding | EVM0031558 | 2.38E-03 | 2.70E-01 |
| Chlorophyll biosynthesis | GSA1 | Aminotransferase class-III | EVM0031578 | 4.01E-02 | 5.24E-02 |
| Chlorophyll biosynthesis | HEMA1 | Shikimate / quinate 5-dehydrogenase | EVM0015686 | 1.89E-01 | 1.35E-02 |
| Chlorophyll biosynthesis | HEMB1 | Delta-aminolevulinic acid dehydratase | EVM0009702 | 1.83E-06 | 1.24E-02 |
| Chlorophyll biosynthesis | HEMB2 | Delta-aminolevulinic acid dehydratase | EVM0004823 | 4.58E-02 | 7.73E-01 |
| Chlorophyll biosynthesis | HEMC | Porphobilinogen deaminase, C-terminal domain | EVM0028353 | 8.94E-18 | 5.28E-09 |
| Chlorophyll biosynthesis | HEMD | Uroporphyrinogen-III synthase HemD | EVM0005875 | 3.71E-01 | 3.03E-02 |
| Chlorophyll biosynthesis | HEME1 | Uroporphyrinogen decarboxylase (URO-D) | EVM0019649 | 1.54E-05 | 4.91E-02 |
| Chlorophyll biosynthesis | HEME3 | Uroporphyrinogen decarboxylase (URO-D) | EVM0012303 | 5.96E-04 | 2.22E-02 |
| Chlorophyll biosynthesis | HEMG2 | Flavin containing amine oxidoreductase | EVM0027844 | 1.13E-03 | 7.29E-01 |
| Chlorophyll biosynthesis | HY2 | Ferredoxin-dependent bilin reductase | EVM0032676 | 3.48E-03 | 9.88E-02 |
| Chlorophyll biosynthesis | PAO | Rieske [2Fe-2S] domain | EVM0023853 | 6.55E-04 | 8.94E-01 |
| Chlorophyll biosynthesis | PORA1 | short chain dehydrogenase | EVM0024685 | 3.40E-01 | 3.17E-03 |
| Chlorophyll biosynthesis | PORA2 | short chain dehydrogenase | EVM0025766 | 4.69E-11 | 9.91E-03 |
| Chlorophyll biosynthesis | PORA3 | short chain dehydrogenase | EVM0031259 | 7.17E-11 | 1.53E-11 |
| Chlorophyll biosynthesis | RCCR | Red chlorophyll catabolite reductase (RCC reductase) | EVM0032481 | 7.63E-03 | 4.85E-01 |

**Table S8. Pigment content in green-leaf *Forsythia* plants after transiently silencing the candidate genes with VIGS.**

| Group | Chl *a* | Chl *b* | Chl *a*+*b* | Carotenoid |
| --- | --- | --- | --- | --- |
| CK (vector control) | 0.57±0.02 a | 0.14±0.01 a | 0.71±0.03 a | 0.30±0.02 a |
| *FsChlH*-silenced | 0.20±0.01 b | 0.05±0.01 b | 0.24±0.01 b | 0.15±0.01 b |
| *FsPOLGAMMA2*-silenced | 0.12±0.02 c | 0.01±0.00 c | 0.12±0.01 c | 0.08±0.00 c |

Total chlorophyll content = Chl *a*+ Chl *b*; For different pigment contents within each column, mean ± se followed by the same alphabetic letters are not significantly different in Duncan’s Multiple Range test under significance level 0.05.

**Table S9. The chloroplast structure in green-leaf *Forsythia* plants after transiently silencing the candidate gene with VIGS.**

| Group | Chloroplast length (μm) | Chloroplast width (μm) | Number of chloroplasts /cell | Thylakoid thickness (μm) | Thylakoids / chloroplast | Number of starch grains / chloroplast |
| --- | --- | --- | --- | --- | --- | --- |
| CK (vector control) | 5.2±1.0b | 2.4±0.2b | 6.4±0.5b | 0.2±0.1a | 20.8±1.5a | 4.6±0.5a |
| *ChlH*-silenced | 6.9±1.6a | 4.6±0.8a | 13.4±2.4a | 0.2±0.1a | 17.6±2.2b | 4.8±1.5a |
| *POLGAMMA2*-silenced | 4.8±1.0b | 2.3±0.4b | 6.7±0.3b | 0.3±0.1a | 13.1±2.6c | 1.2±0.1b |

For each parameter, Mean±SE followed with the different alphabetic letter are significantly different in Duncan’s Multiple Range test under significance level 0.05.

**Table S10. Pigment content in tomato plants after transiently silencing the candidate genes with VIGS.**

| Group | Chl *a* (mg/g) | Chl *b* (mg/g) | Chl *a* + *b* (mg/g) | Carotenoids (mg/g) |
| --- | --- | --- | --- | --- |
| S-CK | 0.35±0.02a | 0.45±0.03a | 0.80±0.05a | 0.13±0.05a |
| S-TRV2 | 0.15±0.03b | 0.22±0.03b | 0.37±0.06b | 0.06±0.02b |
| S-*ChlH*-silenced | 0.07±0.01c | 0.10±0.02c | 0.17±0.03c | 0.04±0.01c |
| S-*POLGAMMA*2-silenced | 0.08±0.02c | 0.02±0.01d | 0.05±0.01d | 0.04±0.01c |

Total chlorophyll content = Chl *a*+ Chl *b*; For different pigment contents within each column, mean ± se followed by the same alphabetic letters are not significantly different in Duncan’s Multiple Range test under significance level 0.05.

**Table S11. Pigment content in tobacco plants after transiently silencing the candidate genes with VIGS.**

| Group | Chl *a* (mg/g) | Chl *b* (mg/g) | Chl *a* + *b* (mg/g) | Carotenoids (mg/g) |
| --- | --- | --- | --- | --- |
| N-CK | 0.22±0.03a | 0.37±0.02a | 0.59±0.05a | 0.10±0.02a |
| N-TRV2 | 0.22±0.03a | 0.37±0.03a | 0.59±0.06a | 0.10±0.02a |
| N-*ChlH*-silenced | 0.13±0.02b | 0.15±0.04b | 0.28±0.05b | 0.06±0.01b |
| N-*POLGAMMA*2-silenced | 0.12±0.04b | 0.03±0.03c | 0.14±0.06c | 0.04±0.02b |

Total chlorophyll content = Chl *a*+ Chl *b*; For different pigment contents within each column, mean ± se followed by the same alphabetic letters are not significantly different in Duncan’s Multiple Range test under significance level 0.05.

**Table S12. The chloroplast structure of tomato leaves with target genes transiently silenced.**

| Sample | Chloroplast length (μm) | Chloroplast width (μm) | Number of chloroplasts /cell | Thylakoid thickness (μm) | Thylakoids / chloroplast | Number of starch grains / chloroplast |
| --- | --- | --- | --- | --- | --- | --- |
| S-CK | 54.67±2.49b | 34.00±1.63b | 8.67±0.94a | 4.33±0.47b | 28.33±1.70b | 0.33±0.47b |
| S-TRV2 | 59±2.16a | 38.00±1.63a | 8.33±0.47a | 5.33±0.47a | 27.67±1.25b | 1.67±0.47a |
| S-*ChlH*-silenced | 60.00±1.63a | 26.00±1.63c | 6.33±1.25b | 2.33±0.47c | 51.67±2.87a | 0.33±0.47b |
| S-*POLGAMMA*2-silenced | 50.67±2.49b | 21.33±0.94d | 7.67±0.94a | / | / | 2.33±0.47a |

For each parameter, Mean±SE followed with the different alphabetic letter are significantly different in Duncan’s Multiple Range test under significance level 0.05.

**Table S13. The chloroplast structure of tobacco leaves with target genes transiently silenced.**

| Sample | Chloroplast length (μm) | Chloroplast width (μm) | Number of chloroplasts /cell | Thylakoid thickness (μm) | Thylakoids / chloroplast | Number of starch grains / chloroplast |
| --- | --- | --- | --- | --- | --- | --- |
| N-CK | 61.33±2.49b | 39.67±2.87a | 11.33±1.25a | 5.00±0.82a | 23.33±2.49a | 5.00±0.82b |
| N-TRV2 | 70.00±1.63a | 36.67±1.89a | 9±0.82b | 5.00±0.82a | 27.67±2.49b | 2.33±0.47a |
| N-*ChlH*-silenced | 52.00±3.27c | 24.00±1.63b | 7.33±1.25c | 3.00±0.82b | 33.00±1.41b | 1.67±0.47a |
| N-*POLGAMMA*2-silenced | 48.67±2.49c | 21.33±3.40b | 6.00±0.82c | / | / | 1.67±0.47a |

For each parameter, Mean±SE followed with the different alphabetic letter are significantly different in Duncan’s Multiple Range test under significance level 0.05.

**Table S14. Pigment content in yellow-leaf Forsythia plants with candidate genes transiently over-expressed.**

| Group | Chl *a* (mg/g) | Chl *b* (mg/g) | Chl *a* + *b* (mg/g) | Carotenoids (mg/g) |
| --- | --- | --- | --- | --- |
| *ChlH-OE* | 5.62±0.1a | 1.17±0.2a | 6.79±0.3a | 1.68±0.2a |
| *ChlH-VC* | 3.76±0.5b | 0.66±0.1b | 4.42±0.2b | 1.6±0.1a |
| *POLGAMMA2-OE* | 5.65±0.4a | 1.74±0.3a | 7.39±0.2a | 1.93±0.3a |
| *POLGAMMA2*-VC | 2.66±0.2b | 0.65±0.1b | 3.31±0.2b | 1.22±0.4a |
| CK | 3.81±0.1b | 0.63±0.3b | 4.44±0.2b | 1.48±0.5a |

Total chlorophyll content = Chl *a*+ Chl *b*; For different pigment contents within each column, mean ± se followed by the same alphabetic letters are not significantly different in Duncan’s Multiple Range test under significance level 0.05.

**Table S15. The chloroplast structure of yellow-leaf Forsythia plants with candidate genes transiently over-expressed.**

| Group | Chloroplast length (μm) | Chloroplast width (μm) | Number of chloroplasts /cell | Thylakoid membrane thickness (μm) | Thylakoids / chloroplast | Number of starch grains / chloroplast |
| --- | --- | --- | --- | --- | --- | --- |
| CK | 50.00±4.32b | 19.33±2.49b | 5.33±0.47a | / | / | 2.67±0.47a |
| Vector control | 52.00±4.32b | 16.67±3.40b | 5.33±0.47a | / | / | 3±0.82a |
| *ChlH-OE* | 58.00±7.48a | 24.00±1.63a | 5.67±0.47a | / | / | 3.33±0.47a |
| *POLGAMMA2-OE* | 64.00±5.89a | 26.33±4.19a | 6.00±0.82a | 2.00±0.82a | 5.33±0.47a | 2.33±0.47a |

For each parameter, Mean±SE followed with the different alphabetic letter are significantly different in Duncan’s Multiple Range test under significance level 0.05.

**Table S16. The GO terms of biological process enriched among genes differentially expressed between *FsChlH*-silenced and vector control plants.**

| GO term | Ontology | Description | P value | FDR |
| --- | --- | --- | --- | --- |
| GO:0015979 | P | photosynthesis | 5.7E-49 | 1.8E-45 |
| GO:0019684 | P | photosynthesis, light reaction | 2.1E-29 | 3.4E-26 |
| GO:0008152 | P | metabolic process | 1.2E-27 | 1.3E-24 |
| GO:0006091 | P | generation of precursor metabolites and energy | 5.5E-24 | 4.5E-21 |
| GO:0009628 | P | response to abiotic stimulus | 2.9E-23 | 1.9E-20 |
| GO:0050896 | P | response to stimulus | 1.5E-22 | 8E-20 |
| GO:0044237 | P | cellular metabolic process | 1.1E-20 | 4.9E-18 |
| GO:0009987 | P | cellular process | 1.5E-20 | 6.2E-18 |
| GO:0034641 | P | cellular nitrogen compound metabolic process | 2.2E-17 | 7.7E-15 |
| GO:0009657 | P | plastid organization | 3.7E-17 | 1.2E-14 |
| GO:0009416 | P | response to light stimulus | 1.4E-16 | 4.2E-14 |
| GO:0009314 | P | response to radiation | 1.8E-16 | 4.9E-14 |
| GO:0009767 | P | photosynthetic electron transport chain | 2.8E-15 | 6.9E-13 |
| GO:0022900 | P | electron transport chain | 1.5E-14 | 3.5E-12 |
| GO:0009266 | P | response to temperature stimulus | 1.3E-13 | 2.7E-11 |
| GO:0009409 | P | response to cold | 2E-12 | 4E-10 |
| GO:0009791 | P | post-embryonic development | 2.4E-12 | 4.6E-10 |
| GO:0009658 | P | chloroplast organization | 5.4E-12 | 9.8E-10 |
| GO:0055114 | P | oxidation reduction | 2.2E-11 | 3.8E-09 |
| GO:0046148 | P | pigment biosynthetic process | 5.3E-11 | 8.6E-09 |
| GO:0018130 | P | heterocycle biosynthetic process | 6.2E-11 | 9.6E-09 |
| GO:0042180 | P | cellular ketone metabolic process | 8E-11 | 1.2E-08 |
| GO:0033014 | P | tetrapyrrole biosynthetic process | 1.7E-10 | 2.4E-08 |
| GO:0006779 | P | porphyrin biosynthetic process | 2.3E-10 | 3.1E-08 |
| GO:0009058 | P | biosynthetic process | 3.4E-10 | 4.4E-08 |
| GO:0005975 | P | carbohydrate metabolic process | 4.3E-10 | 5.4E-08 |
| GO:0044262 | P | cellular carbohydrate metabolic process | 9.2E-10 | 1.1E-07 |
| GO:0042440 | P | pigment metabolic process | 1.4E-09 | 1.6E-07 |
| GO:0043436 | P | oxoacid metabolic process | 1.5E-09 | 1.7E-07 |
| GO:0019752 | P | carboxylic acid metabolic process | 1.5E-09 | 1.7E-07 |
| GO:0006082 | P | organic acid metabolic process | 1.6E-09 | 1.7E-07 |
| GO:0051188 | P | cofactor biosynthetic process | 4.7E-09 | 4.8E-07 |
| GO:0006950 | P | response to stress | 6.3E-09 | 6.2E-07 |
| GO:0015995 | P | chlorophyll biosynthetic process | 6.7E-09 | 6.4E-07 |
| GO:0033013 | P | tetrapyrrole metabolic process | 7.6E-09 | 7E-07 |
| GO:0005982 | P | starch metabolic process | 1E-08 | 9.5E-07 |
| GO:0051186 | P | cofactor metabolic process | 1.2E-08 | 1.1E-06 |
| GO:0005976 | P | polysaccharide metabolic process | 1.3E-08 | 1.1E-06 |
| GO:0044249 | P | cellular biosynthetic process | 1.5E-08 | 1.3E-06 |
| GO:0006778 | P | porphyrin metabolic process | 2E-08 | 1.6E-06 |
| GO:0009773 | P | photosynthetic electron transport in photosystem I | 2.6E-08 | 2E-06 |
| GO:0046483 | P | heterocycle metabolic process | 3.9E-08 | 3E-06 |
| GO:0006519 | P | cellular amino acid and derivative metabolic process | 6.1E-08 | 4.6E-06 |
| GO:0044238 | P | primary metabolic process | 9.3E-08 | 6.8E-06 |
| GO:0006461 | P | protein complex assembly | 9.9E-08 | 7E-06 |
| GO:0070271 | P | protein complex biogenesis | 9.9E-08 | 7E-06 |
| GO:0042221 | P | response to chemical stimulus | 1.1E-07 | 7.3E-06 |
| GO:0044271 | P | cellular nitrogen compound biosynthetic process | 2.1E-07 | 1.4E-05 |
| GO:0015994 | P | chlorophyll metabolic process | 3E-07 | 0.00002 |
| GO:0010876 | P | lipid localization | 3.1E-07 | 0.00002 |
| GO:0006073 | P | cellular glucan metabolic process | 3.3E-07 | 2.1E-05 |
| GO:0044042 | P | glucan metabolic process | 4.3E-07 | 2.7E-05 |
| GO:0009765 | P | photosynthesis, light harvesting | 5.5E-07 | 3.4E-05 |
| GO:0006725 | P | cellular aromatic compound metabolic process | 7E-07 | 4.2E-05 |
| GO:0010207 | P | photosystem II assembly | 1.4E-06 | 8.4E-05 |
| GO:0048856 | P | anatomical structure development | 1.6E-06 | 9.3E-05 |
| GO:0015977 | P | carbon fixation | 1.6E-06 | 9.4E-05 |
| GO:0010027 | P | thylakoid membrane organization | 1.8E-06 | 0.0001 |
| GO:0009668 | P | plastid membrane organization | 1.8E-06 | 0.0001 |
| GO:0009642 | P | response to light intensity | 2.2E-06 | 0.00011 |
| GO:0044085 | P | cellular component biogenesis | 2.1E-06 | 0.00011 |
| GO:0044264 | P | cellular polysaccharide metabolic process | 2.5E-06 | 0.00013 |
| GO:0008610 | P | lipid biosynthetic process | 2.5E-06 | 0.00013 |
| GO:0032787 | P | monocarboxylic acid metabolic process | 2.8E-06 | 0.00014 |
| GO:0016109 | P | tetraterpenoid biosynthetic process | 3.3E-06 | 0.00016 |
| GO:0016117 | P | carotenoid biosynthetic process | 3.3E-06 | 0.00016 |
| GO:0010109 | P | regulation of photosynthesis | 3.3E-06 | 0.00016 |
| GO:0048513 | P | organ development | 3.9E-06 | 0.00018 |
| GO:0032501 | P | multicellular organismal process | 3.9E-06 | 0.00018 |
| GO:0048731 | P | system development | 4E-06 | 0.00019 |
| GO:0007275 | P | multicellular organismal development | 4.8E-06 | 0.00022 |
| GO:0045038 | P | protein import into chloroplast thylakoid membrane | 6.4E-06 | 0.00029 |
| GO:0016043 | P | cellular component organization | 6.4E-06 | 0.00029 |
| GO:0043623 | P | cellular protein complex assembly | 6.6E-06 | 0.00029 |
| GO:0009644 | P | response to high light intensity | 9E-06 | 0.00039 |
| GO:0032502 | P | developmental process | 9.1E-06 | 0.00039 |
| GO:0005996 | P | monosaccharide metabolic process | 9.6E-06 | 0.0004 |
| GO:0042548 | P | regulation of photosynthesis, light reaction | 1.1E-05 | 0.00044 |
| GO:0016051 | P | carbohydrate biosynthetic process | 1.4E-05 | 0.00058 |
| GO:0010035 | P | response to inorganic substance | 1.6E-05 | 0.00064 |
| GO:0034614 | P | cellular response to reactive oxygen species | 1.6E-05 | 0.00064 |
| GO:0051234 | P | establishment of localization | 1.8E-05 | 0.00071 |
| GO:0043933 | P | macromolecular complex subunit organization | 1.8E-05 | 0.00072 |
| GO:0008299 | P | isoprenoid biosynthetic process | 1.9E-05 | 0.00072 |
| GO:0016108 | P | tetraterpenoid metabolic process | 0.00002 | 0.00074 |
| GO:0006575 | P | cellular amino acid derivative metabolic process | 0.00002 | 0.00074 |
| GO:0000302 | P | response to reactive oxygen species | 0.00002 | 0.00074 |
| GO:0016116 | P | carotenoid metabolic process | 0.00002 | 0.00074 |
| GO:0045036 | P | protein targeting to chloroplast | 2.1E-05 | 0.00076 |
| GO:0006066 | P | alcohol metabolic process | 2.4E-05 | 0.00085 |
| GO:0034599 | P | cellular response to oxidative stress | 2.4E-05 | 0.00085 |
| GO:0065003 | P | macromolecular complex assembly | 2.6E-05 | 0.00091 |
| GO:0043467 | P | regulation of generation of precursor metabolites and energy | 2.7E-05 | 0.00094 |
| GO:0044106 | P | cellular amine metabolic process | 2.7E-05 | 0.00094 |
| GO:0006629 | P | lipid metabolic process | 2.8E-05 | 0.00096 |
| GO:0009886 | P | post-embryonic morphogenesis | 3.1E-05 | 0.001 |
| GO:0016052 | P | carbohydrate catabolic process | 3.3E-05 | 0.0011 |
| GO:0051179 | P | localization | 3.3E-05 | 0.0011 |
| GO:0000272 | P | polysaccharide catabolic process | 3.6E-05 | 0.0012 |
| GO:0019252 | P | starch biosynthetic process | 3.6E-05 | 0.0012 |
| GO:0006810 | P | transport | 3.9E-05 | 0.0013 |
| GO:0019253 | P | reductive pentose-phosphate cycle | 4.7E-05 | 0.0015 |
| GO:0006979 | P | response to oxidative stress | 0.00005 | 0.0016 |
| GO:0009624 | P | response to nematode | 5.4E-05 | 0.0017 |
| GO:0009607 | P | response to biotic stimulus | 6.1E-05 | 0.0019 |
| GO:0006418 | P | tRNA aminoacylation for protein translation | 6.8E-05 | 0.0021 |
| GO:0043038 | P | amino acid activation | 6.8E-05 | 0.0021 |
| GO:0043039 | P | tRNA aminoacylation | 6.8E-05 | 0.0021 |
| GO:0043155 | P | negative regulation of photosynthesis, light reaction | 7.4E-05 | 0.0021 |
| GO:0010205 | P | photoinhibition | 7.4E-05 | 0.0021 |
| GO:0019685 | P | photosynthesis, dark reaction | 7.4E-05 | 0.0021 |
| GO:0009605 | P | response to external stimulus | 8.4E-05 | 0.0024 |
| GO:0006520 | P | cellular amino acid metabolic process | 8.8E-05 | 0.0025 |
| GO:0009308 | P | amine metabolic process | 9.3E-05 | 0.0027 |
| GO:0009768 | P | photosynthesis, light harvesting in photosystem I | 0.00011 | 0.003 |
| GO:0051707 | P | response to other organism | 0.00011 | 0.003 |
| GO:0016123 | P | xanthophyll biosynthetic process | 0.00011 | 0.003 |
| GO:0022607 | P | cellular component assembly | 0.00011 | 0.003 |
| GO:0019438 | P | aromatic compound biosynthetic process | 0.00014 | 0.0038 |
| GO:0015837 | P | amine transport | 0.00015 | 0.0042 |
| GO:0016119 | P | carotene metabolic process | 0.00016 | 0.0043 |
| GO:0016120 | P | carotene biosynthetic process | 0.00016 | 0.0043 |
| GO:0015849 | P | organic acid transport | 0.00019 | 0.0049 |
| GO:0046942 | P | carboxylic acid transport | 0.00019 | 0.0049 |
| GO:0016114 | P | terpenoid biosynthetic process | 0.00019 | 0.0049 |
| GO:0019748 | P | secondary metabolic process | 0.00022 | 0.0056 |
| GO:0006720 | P | isoprenoid metabolic process | 0.00024 | 0.0061 |
| GO:0010051 | P | xylem and phloem pattern formation | 0.00025 | 0.0064 |
| GO:0044275 | P | cellular carbohydrate catabolic process | 0.00026 | 0.0065 |
| GO:0042398 | P | cellular amino acid derivative biosynthetic process | 0.00027 | 0.0068 |
| GO:0019318 | P | hexose metabolic process | 0.00028 | 0.0069 |
| GO:0000271 | P | polysaccharide biosynthetic process | 0.00028 | 0.0069 |
| GO:0009793 | P | embryonic development ending in seed dormancy | 0.00035 | 0.0085 |
| GO:0009698 | P | phenylpropanoid metabolic process | 0.00038 | 0.0091 |
| GO:0044255 | P | cellular lipid metabolic process | 0.00038 | 0.0092 |
| GO:0034621 | P | cellular macromolecular complex subunit organization | 0.0004 | 0.0096 |
| GO:0006996 | P | organelle organization | 0.00042 | 0.0099 |
| GO:0034637 | P | cellular carbohydrate biosynthetic process | 0.00042 | 0.01 |
| GO:0010114 | P | response to red light | 0.00045 | 0.01 |
| GO:0048316 | P | seed development | 0.00048 | 0.011 |
| GO:0042542 | P | response to hydrogen peroxide | 0.00048 | 0.011 |
| GO:0006865 | P | amino acid transport | 0.00054 | 0.012 |
| GO:0010154 | P | fruit development | 0.00062 | 0.014 |
| GO:0042592 | P | homeostatic process | 0.00063 | 0.014 |
| GO:0034622 | P | cellular macromolecular complex assembly | 0.00064 | 0.014 |
| GO:0009251 | P | glucan catabolic process | 0.00071 | 0.016 |
| GO:0005983 | P | starch catabolic process | 0.00071 | 0.016 |
| GO:0009637 | P | response to blue light | 0.00073 | 0.016 |
| GO:0007167 | P | enzyme linked receptor protein signaling pathway | 0.00076 | 0.016 |
| GO:0007169 | P | transmembrane receptor protein tyrosine kinase signaling pathway | 0.00076 | 0.016 |
| GO:0046246 | P | terpene biosynthetic process | 0.00075 | 0.016 |
| GO:0006633 | P | fatty acid biosynthetic process | 0.00076 | 0.016 |
| GO:0006399 | P | tRNA metabolic process | 0.00081 | 0.017 |
| GO:0010033 | P | response to organic substance | 0.00083 | 0.017 |
| GO:0046394 | P | carboxylic acid biosynthetic process | 0.00089 | 0.018 |
| GO:0016053 | P | organic acid biosynthetic process | 0.00089 | 0.018 |
| GO:0016122 | P | xanthophyll metabolic process | 0.00091 | 0.019 |
| GO:0009744 | P | response to sucrose stimulus | 0.00097 | 0.02 |
| GO:0006631 | P | fatty acid metabolic process | 0.001 | 0.021 |
| GO:0009790 | P | embryonic development | 0.001 | 0.021 |
| GO:0048608 | P | reproductive structure development | 0.0011 | 0.022 |
| GO:0003006 | P | reproductive developmental process | 0.0011 | 0.022 |
| GO:0044247 | P | cellular polysaccharide catabolic process | 0.0011 | 0.023 |
| GO:0034285 | P | response to disaccharide stimulus | 0.0012 | 0.024 |
| GO:0042214 | P | terpene metabolic process | 0.0014 | 0.027 |
| GO:0006612 | P | protein targeting to membrane | 0.0015 | 0.029 |
| GO:0022414 | P | reproductive process | 0.0015 | 0.03 |
| GO:0046351 | P | disaccharide biosynthetic process | 0.0015 | 0.03 |
| GO:0000003 | P | reproduction | 0.0017 | 0.032 |
| GO:0009110 | P | vitamin biosynthetic process | 0.0017 | 0.032 |
| GO:0009853 | P | photorespiration | 0.0017 | 0.033 |
| GO:0045426 | P | quinone cofactor biosynthetic process | 0.0018 | 0.033 |
| GO:0042375 | P | quinone cofactor metabolic process | 0.0018 | 0.033 |
| GO:0051704 | P | multi-organism process | 0.0019 | 0.036 |
| GO:0007166 | P | cell surface receptor linked signaling pathway | 0.002 | 0.037 |
| GO:0006775 | P | fat-soluble vitamin metabolic process | 0.0021 | 0.038 |
| GO:0042362 | P | fat-soluble vitamin biosynthetic process | 0.0021 | 0.038 |
| GO:0009250 | P | glucan biosynthetic process | 0.0021 | 0.039 |
| GO:0033036 | P | macromolecule localization | 0.0021 | 0.039 |
| GO:0009699 | P | phenylpropanoid biosynthetic process | 0.0022 | 0.039 |
| GO:0006721 | P | terpenoid metabolic process | 0.0023 | 0.04 |
| GO:0019725 | P | cellular homeostasis | 0.0022 | 0.04 |
| GO:0015833 | P | peptide transport | 0.0023 | 0.041 |
| GO:0006857 | P | oligopeptide transport | 0.0023 | 0.041 |
| GO:0009312 | P | oligosaccharide biosynthetic process | 0.0025 | 0.044 |
| GO:0065008 | P | regulation of biological quality | 0.0025 | 0.044 |
| GO:0009639 | P | response to red or far red light | 0.0025 | 0.044 |

**Table S17. The GO biological process enriched among genes differentially expressed between *FsPOLGAMMA2*-silenced and vector control plants.**

| GO term | Ontology | Description | P value | FDR |
| --- | --- | --- | --- | --- |
| GO:0009698 | P | phenylpropanoid metabolic process | 7.70E-08 | 8.90E-06 |
| GO:0019748 | P | secondary metabolic process | 1.90E-06 | 8.40E-05 |
| GO:0006575 | P | cellular amino acid derivative metabolic process | 2.20E-06 | 8.40E-05 |
| GO:0009611 | P | response to wounding | 4.30E-06 | 0.00012 |
| GO:0006725 | P | cellular aromatic compound metabolic process | 8.30E-06 | 0.00019 |
| GO:0006979 | P | response to oxidative stress | 5.00E-05 | 0.00095 |
| GO:0009605 | P | response to external stimulus | 0.00016 | 0.0023 |
| GO:0006519 | P | cellular amino acid and derivative metabolic process | 0.00016 | 0.0023 |
| GO:0006950 | P | response to stress | 0.00034 | 0.0043 |
| GO:0009314 | P | response to radiation | 0.00082 | 0.0075 |
| GO:0009416 | P | response to light stimulus | 0.00072 | 0.0075 |
| GO:0044255 | P | cellular lipid metabolic process | 0.00085 | 0.0075 |
| GO:0050896 | P | response to stimulus | 0.00067 | 0.0075 |
| GO:0065008 | P | regulation of biological quality | 0.0012 | 0.0096 |
| GO:0009628 | P | response to abiotic stimulus | 0.0017 | 0.013 |
| GO:0042221 | P | response to chemical stimulus | 0.003 | 0.021 |
| GO:0006629 | P | lipid metabolic process | 0.0032 | 0.022 |
| GO:0009987 | P | cellular process | 0.0038 | 0.024 |
